# PIP5K1A Suppresses Ferroptosis and Induces Sorafenib Resistance by Stabilizing NRF2 in Hepatocellular Carcinoma

**DOI:** 10.1002/advs.202504372

**Published:** 2025-05-23

**Authors:** Mengzhou Guo, Sinuo Chen, Jialei Sun, Ruchen Xu, Zhuoran Qi, Jie Li, Lianer Zhou, Yuan Fang, Tianshu Liu, Jinglin Xia

**Affiliations:** ^1^ Department of Medical Oncology Zhongshan Hospital Fudan University 180 Fenglin Road Shanghai 200032 China; ^2^ Liver Cancer Institute and Key Laboratory of Carcinogenesis and Cancer Invasion (Ministry of Education) Zhongshan Hospital Fudan University 180 Fenglin Road Shanghai 200032 China; ^3^ Department of Gastroenterology and Hepatology and Shanghai Institute of Liver Diseases Zhongshan Hospital Fudan University 180 Fenglin Road Shanghai 200032 China; ^4^ Department of Liver Surgery Zhongshan Hospital Fudan University 180 Fenglin Road Shanghai 200032 China

**Keywords:** ferroptosis, hepatocellular carcinoma, NRF2, PIP5K1A, sorafenib resistance

## Abstract

Hepatocellular carcinoma (HCC) is a leading cause of cancer‐related mortality worldwide. Ferroptosis, an iron‐dependent form of programmed cell death driven by lipid peroxidation, has emerged as a promising strategy for cancer treatment. However, the development of ferroptosis resistance limits the efficacy of such treatments. This study reports that phosphatidylinositol 4‐phosphate 5‐kinase 1 alpha (PIP5K1A) promotes HCC tumorigenesis and predicts poor prognosis in HCC patients. Knockdown of PIP5K1A enhances lipid peroxidation and increases sensitivity to sorafenib‐induced ferroptosis by inhibiting the activation of downstream ferroptosis‐related genes regulated by nuclear factor erythroid‐2‐related factor 2 (NRF2). Mechanistically, PIP5K1A competitively binds to the Kelch domain of Kelch‐like ECH‐associated protein 1 in a kinase‐independent manner, leading to NRF2 escaping from ubiquitination degradation, thereby promoting NRF2‐dependent transcription and suppressing ferroptosis. Furthermore, ISA‐2011B, a PIP5K1A‐specific inhibitor, effectively inhibits HCC growth and sensitized HCC cells to sorafenib. In conclusion, PIP5K1A is a promising therapeutic target for improving the efficacy of sorafenib and other ferroptosis inducers in HCC.

## Graphical abstract

Inducing ferroptosis is a promising strategy for combating tumor resistance. Upregulated PIP5K1A competes with NRF2 for KEAP1 binding, which decreases the ubiquitination degradation of NRF2 and promotes its nuclear translocation, thereby suppressing ferroptosis, and ultimately driving hepatocellular carcinoma (HCC) tumorigeneses and sorafenib resistance. Thus, targeting PIP5K1A can be a promising approach to inhibit HCC progression and overcome sorafenib resistance.

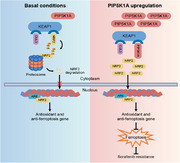

## 1 Introduction

Hepatocellular carcinoma (HCC) is the most prevalent liver cancer subtype and the third leading cause of cancer‐related mortality worldwide.^[^ ^1^ ^]^ Due to the insidious onset of HCC, most patients are initially diagnosed at an advanced stage, with poor prognosis and ineligibility for curative local treatment.^[^ ^2^ ^]^ Although the combination of anti‐angiogenic agents and immunotherapy has entered a golden age of rapid development, some patients cannot undergo this combined treatment due to adverse effects or contraindications to immunotherapy. Currently, multi‐tyrosine kinase inhibitors (mTKIs) are the cornerstone of treatment for advanced HCC. However, the clinical efficacy of these drugs is limited due to the development of drug resistance. Therefore, it is crucial to elucidate the mechanisms underlying drug resistance and identify novel therapeutic targets for HCC.

Sorafenib, a mTKI that inhibits the proliferation and angiogenesis of tumor cells by blocking the activities of Raf, vascular endothelial growth factor receptors 2 and 3, platelet‐derived growth factor receptors, and c‐kit signaling,^[^ ^3^ ^]^ was approved by the Food and Drug Administration as the first and consistently certified first‐line treatment for advanced HCC.^[^ ^4^, ^5^ ^]^ However, sorafenib only prolongs the median survival time of patients by merely three months compared to placebo, primarily due to acquired drug resistance after six months of treatment.^[^ ^6^ ^]^ Notably, sorafenib has recently been recognized as an inducer of ferroptosis, a newly described form of regulated cell death. Triggering ferroptosis can resensitize HCC cells to sorafenib, offering a potential strategy to overcome sorafenib resistance.^[^ ^7^ ^]^

Distinct from apoptosis, necrosis, and autophagy, ferroptosis is an iron‐dependent form of cell death characterized by oxidative stress, intracellular iron accumulation, and lipid reactive oxygen species (ROS) production.^[^ ^8^ ^]^ Elucidating the molecular mechanisms underlying ferroptosis offers a promising therapeutic strategy, particularly for reversing drug resistance in cancer treatment. Ferroptosis is tightly regulated by complex cellular metabolic pathways that involve imbalances in redox homeostasis, iron handling, and lipid metabolism. Nuclear factor E2‐related factor 2 (NRF2) is the master transcriptional regulator of antioxidant gene network.^[^ ^9^ ^]^ Under basal conditions, NRF2 binds to Kelch‐like ECH‐associated protein 1 (KEAP1) via the ETGE and DLG motifs.^[^ ^10^, ^11^ ^]^ KEAP1 acts as a substrate adaptor for the cullin3 (Cul3)‐dependent E3 ubiquitin ligase complex, promoting NRF2 ubiquitination and subsequent degradation in the cytoplasm. Under stress conditions, KEAP1 undergoes cysteine modification, leading to its conformational change that release NRF2. Consequently, NRF2 translocates to the nucleus, binds to the antioxidant responsive elements (AREs) in the promoter region of target genes, and activates their transcription. These genes are crucial for the metabolic pathways associated with ferroptosis.^[^ ^9^ ^]^ Thus, targeting KEAP1‐NRF2 axis is a promising therapeutic strategy for overcoming resistance to sorafenib‐induced ferroptosis.

Phosphatidylinositol 4‐phosphate 5‐kinase 1 alpha (PIP5K1A) is a type I phosphatidylinositol phosphate kinase that catalyzes phosphatidylinositol‐4‐phosphate (PI4P) into phosphatidylinositol‐4,5‐bisphosphate (PIP2).^[^ ^12^ ^]^ PIP2, a major member of the phosphoinositide family, acts as a secondary messenger that regulates diverse biological processes. As a substrate for the production of phosphatidylinositol 3,4,5‐triphosphate (PIP3), PIP2 is essential for activating the PI3K/AKT pathway.^[^ ^13^ ^]^ To the best of our knowledge, PIP5K1A has only been implicated in liver regeneration after liver injury,^[^ ^14^ ^]^ its role in HCC remains largely unexplored. In this study, we demonstrate that PIP5K1A disrupts the KEAP1‐NRF2 interaction, stabilizing NRF2 and thereby suppressing ferroptosis and promoting sorafenib resistance in HCC cells. These findings suggest that targeting PIP5K1A could increase the susceptibility of HCC cells to ferroptosis and represent a potential therapeutic strategy for cancer treatment.

## 2 Results

### 2.1 PIP5K1A Expression Is Upregulated in HCC and Correlates with a Poor Prognosis

**Figure 1:**
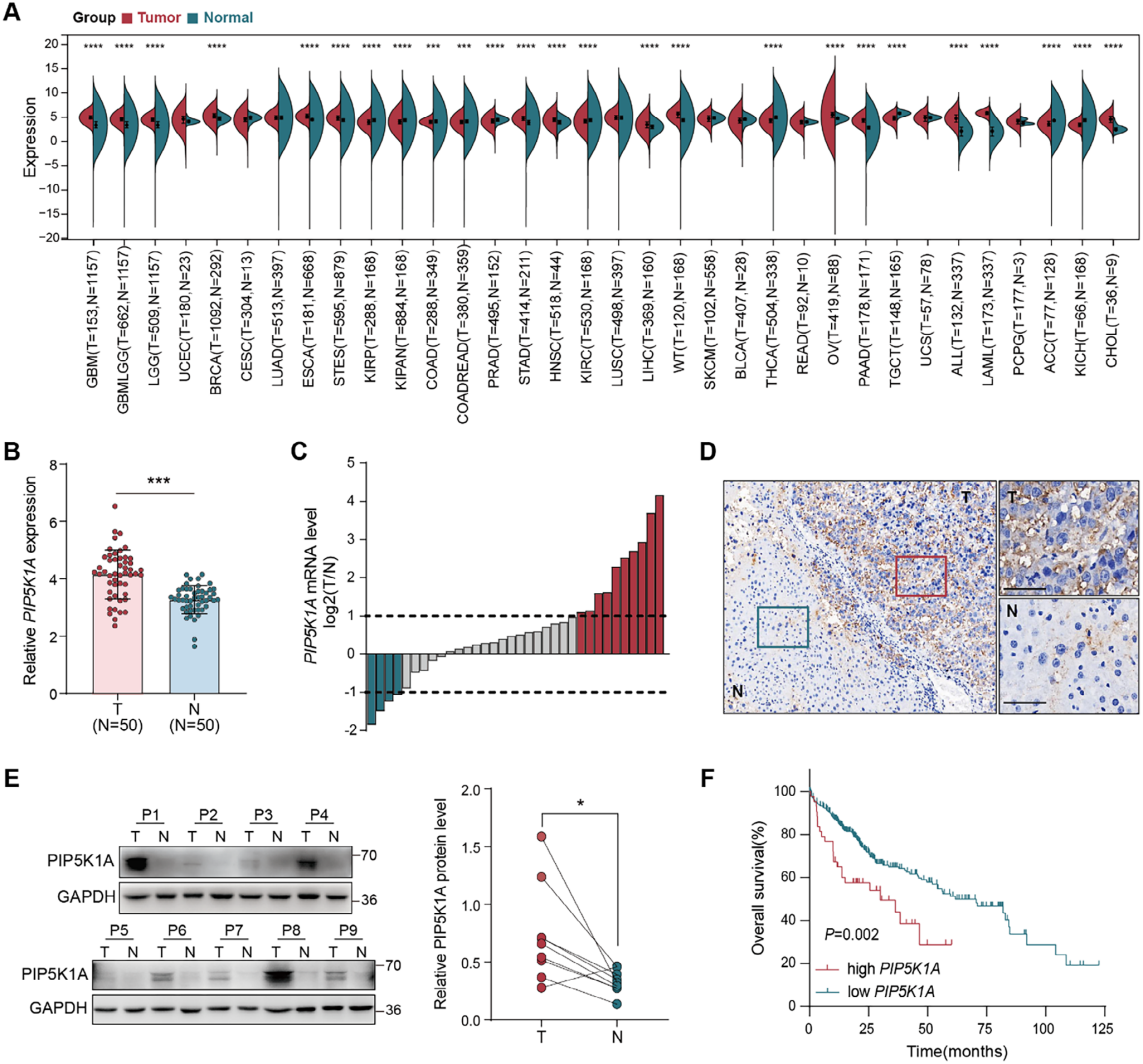
PIP5K1A is upregulated and associated with poor prognosis in HCC. A) *PIP5K1A* mRNA levels of tumor and adjacent normal tissues in pan‐cancer from TCGA cohort. B) *PIP5K1A* mRNA levels in 50 paired tumor and adjacent normal tissues from the TCGA HCC cohort. C) RNA extraction and qRT‐PCRanalysis of *PIP5K1A* mRNA expression in 34 paired tumor and nontumor liver tissues from Zhongshan Hospital cohort. D) Representative IHC staining for PIP5K1A in a spontaneous HCC model driven by c‐Myc. Scale bar: 50 µm. E) Western blotting of PIP5K1A protein expression in nine paired tumor and nontumor liver tissues. F) Kaplan–Meier curves for overall survival according to *PIP5K1A* mRNA expression in HCC specimens form the TCGA cohort. *p < 0.05, **p < 0.01, ***p < 0.001, ****p < 0.0001. Abbreviations: HCC: hepatocellular carcinoma; T: tumor tissues; N: nontumor liver tissues; IHC: Immunohistochemistry; qRT‐PCR: quantitative real‐time polymerase chain reaction.

To evaluate the expression and potential role of PIP5K1A in HCC, we analyzed a publicly available TCGA dataset. As shown in **Figure** **1**A, *PIP5K1A* expression was significantly upregulated in solid tumors of the gastroenterological and hepatobiliary systems. Further analysis of HCC revealed that *PIP5K1A* mRNA expression was significantly higher in tumor tissues than that in paired normal tissue (Figure 1B). In addition, we confirmed that *PIP5K1A* mRNA expression was upregulated in 73.5% (25/34) of the HCC tissues in our cohort (Figure 1C). To further investigate PIP5K1A protein levels, we performed immunohistochemistry (IHC) analysis in a spontaneous HCC model driven by c‐Myc, and western blotting in nine pairs of fresh HCC and adjacent normal tissues. PIP5K1A protein levels were significantly higher in tumor tissues than that in normal liver tissues, which was consistent with the mRNA expression (Figure 1D,E). Furthermore, survival analysis of TCGA cohort revealed that high *PIP5K1A* expression was significantly associated with shorter overall survival (OS) (Figure 1F). Taken together, these results demonstrate that PIP5K1A expression is aberrantly increased in HCC and may serve as a predictor of poor prognosis.

### 2.2 PIP5K1A Promotes HCC Growth In Vitro and In Vivo

**Figure 2:**
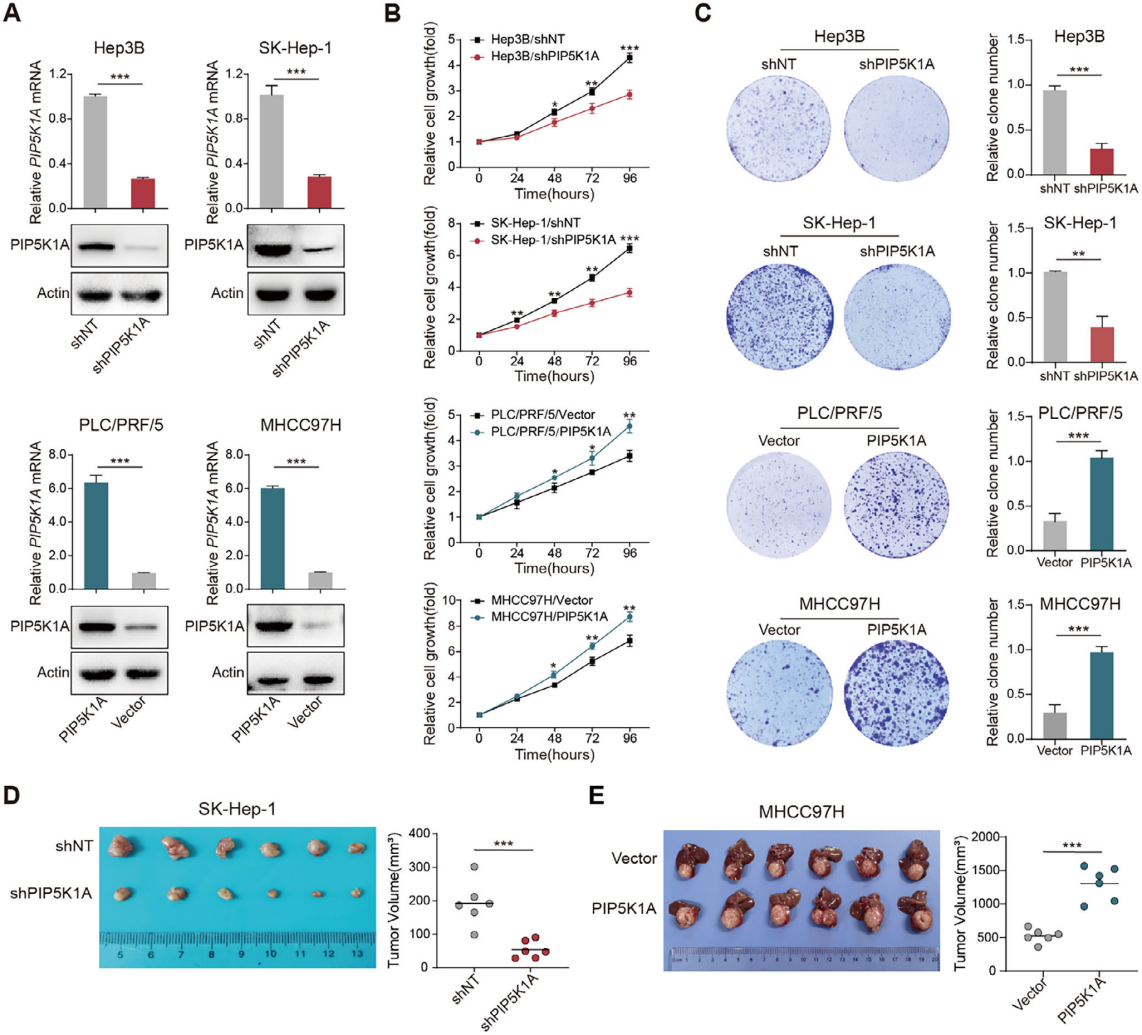
PIP5K1A promotes HCC growth in vitro and in vivo. A) The knockdown and overexpression efficiency of PIP5K1A was validated at the protein and mRNA levels in HCC cells. B) Effects of PIP5K1A knockdown and overexpression on cell proliferation were assessed using CCK8 assay (*n* = 3). C) Effects of PIP5K1A knockdown and overexpression on cell proliferation were determined by colony formation assay (*n* = 3). D) Effects of PIP5K1A knockdown on HCC tumor growth in the subcutaneous xenograft mouse model. Tumor volumes are shown in the right panel (*n* = 6). E) Effects of PIP5K1A overexpression on HCC tumor growth in the orthotopic implantation mouse model. Tumor volumes are shown in the right panel (*n* = 6). Data are presented as the mean ± SD. *p < 0.05, **p < 0.01, ***p < 0.001. Abbreviations: HCC: hepatocellular carcinoma; CCK8: Cell Counting Kit‐8.

To investigate the oncogenic role of PIP5K1A in HCC, we examined its endogenous expression in various HCC cell lines. Due to the relatively high expression of PIP5K1A in Hep3B and SK‐Hep‐1 cells, these two cell lines were selected for PIP5K1A silencing experiments. Conversely, MHCC97H and PLC/PRF/5 cells, which exhibited relatively low PIP5K1A levels, were used for PIP5K1A overexpression analyses (Figure S1, Supporting Information). The efficiency of PIP5K1A overexpression and knockdown was confirmed at the both mRNA and protein levels compared to negative controls (**Figure** **2**A). Next, we explored the role of PIP5K1A in the regulation of HCC cell growth. PIP5K1A knockdown markedly reduced the proliferation rate and colony formation ability of Hep3B and SK‐Hep‐1 cells, whereas PIP5K1A overexpression in PLC/PRF/5 and MHCC97H cells significantly enhanced their proliferation and clonogenicity (Figure 2B,C).

To further verify the effects of PIP5K1A on HCC growth in vivo, we established cell‐derived xenograft models and found that PIP5K1A knockdown markedly inhibited HCC growth (Figure 2D and Figure S2A, Supporting Information). In addition, using orthotopic liver xenograft tumor models, we demonstrated that PIP5K1A overexpression significantly promoted liver tumor growth (Figure 2E). Taken together, these findings indicate that PIP5K1A plays a critical role in promoting HCC cell proliferation.

### 2.3 PIP5K1A Disrupts Redox Homoeostasis and Inhibits Ferroptosis in HCC

**Figure 3:**
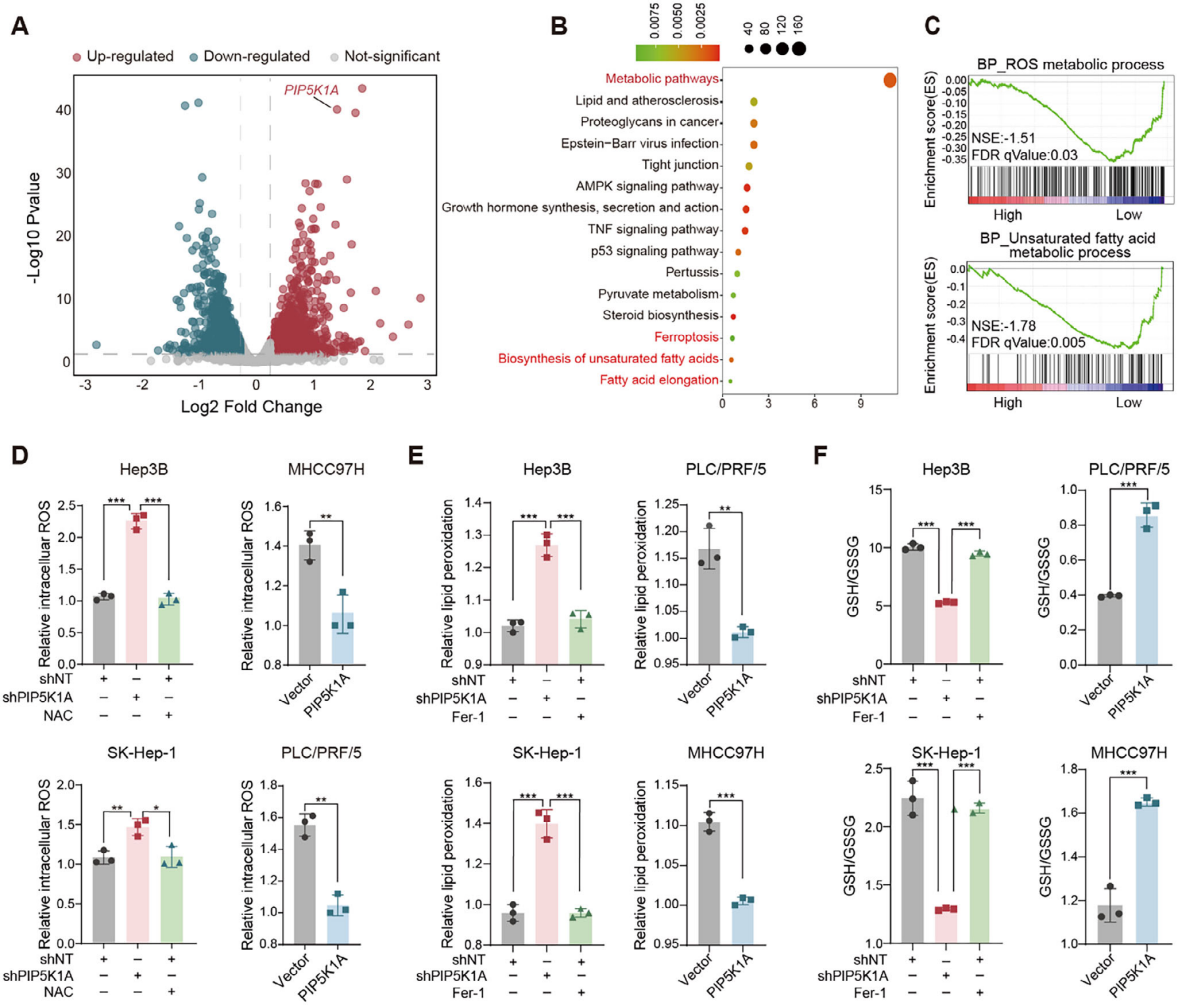
PIP5K1A disrupts redox homoeostasis and inhibits ferroptosis in HCC. A) Volcano plots of DEGs between the Hep3B/shPIP5K1A and Hep3B/shNT group. The criterion for identification of DEGs is |Fold change| >1.2 and FDR < 0.05 (*n* = 3). B) KEGG enrichment analysis of DEGs. The top 15 enriched pathways were summarized. The number of genes represented in each pathway was indicated by point size, and the *p*‐value was shown by point color. C) GSEA enrichment analysis showed that ROS and unsaturated fatty acid metabolic process were activated in the group with low *PIP5K1A* expression. The high and low groups of PIP5K1A were classified by the median expression of *PIP5K1A* in HCC specimens form the TCGA cohort. D) Total ROS levels in the indicated cells were stained by CM‐H2DCFDA and measured by flow cytometry upon treatment with control or NAC (5 × 10^−3^ m) for 24 h (*n* = 3). E) Lipid ROS levels in the indicated cells were stained by C11‐BODIPY and determined using flow cytometry upon treatment with control or Fer‐1(10 µm) for 24 h (*n* = 3). F) The GSH/GSSG ratio in the indicated cells was measured upon treatment with control or Fer‐1(10 µm) for 24 h (*n* = 3). Data are presented as the mean ± SD. *p < 0.05, **p < 0.01, ***p < 0.001. Abbreviations: HCC: hepatocellular carcinoma; DEGs: differentially expressed genes; KEGG: Kyoto Encyclopedia of Genes and Genomes; GSEA: Gene set enrichment analysis; ROS: reactive oxygen species; NAC: N‐acetyl‐l‐cysteine; Fer‐1: Ferrostatin‐1.

To explore the molecular mechanism by which PIP5K1A promotes HCC progression, we performed RNA sequencing to identify differentially expressed genes (DEGs) between Hep3B cells with stable PIP5K1A knockdown and control groups (**Figure** **3**A). Compared to controls, PIP5K1A knockdown resulted in 1388 genes upregulated and 1294 genes downregulated. Kyoto Encyclopedia of Genes and Genomes (KEGG) analysis of these DEGs revealed enrichment in ferroptosis‐related pathways, such as lipid metabolism, including the biosynthesis of unsaturated fatty acids and fatty acid elongation (Figure 3B). In addition, we stratified TCGA cohort samples into high and low *PIP5K1A* expression groups based on median expression. Gene set enrichment analysis (GSEA) indicated that ROS and unsaturated fatty acids metabolic processes were activated in the low *PIP5K1A* expression group (Figure 3C). These findings suggest that PIP5K1A is involved in the regulation of redox homeostasis and ferroptosis.

We next focused on the effects of PIP5K1A on redox homeostasis. PIP5K1A knockdown in Hep3B and SK‐Hep‐1 cells significantly increased total ROS levels compared to control groups, an effect could reverse by the antioxidant N‐acetyl‐l‐cysteine. In contrast, PIP5K1A overexpression markedly reduced total ROS levels in HCC cells compared with that in controls (Figure 3D). Since mitochondria ROS is a major component of endogenic ROS, we also measured mitochondrial ROS levels, and found that PIP5K1A knockdown or overexpression could significantly increase or decrease the levels of mitochondrial ROS as compared with controls, respectively (Figure S2B, Supporting Information).

Ferroptosis is triggered by redox imbalance, with lipid ROS and GSH/GSSG ratio serving as well‐known markers. As shown in Figure 3E,F, PIP5K1A knockdown significantly increased lipid peroxidation levels and decreased the GSH/GSSG ratio compared with those in controls, which was reversed by the ferroptosis inhibitor Ferrostatin‐1 (Fer‐1). In contrast, overexpression of PIP5K1A in PLC/PRF/5 and MHCC97H cells markedly reduced lipid peroxidation levels and elevated the GSH/GSSG ratio compared with that in controls. Taken together, these results demonstrate that PIP5K1A is involved in regulating redox homeostasis and ferroptosis.

### 2.4 PIP5K1A Protects the HCC Cells from Sorafenib‐Induced Death by Inhibiting Ferroptosis

**Figure 4:**
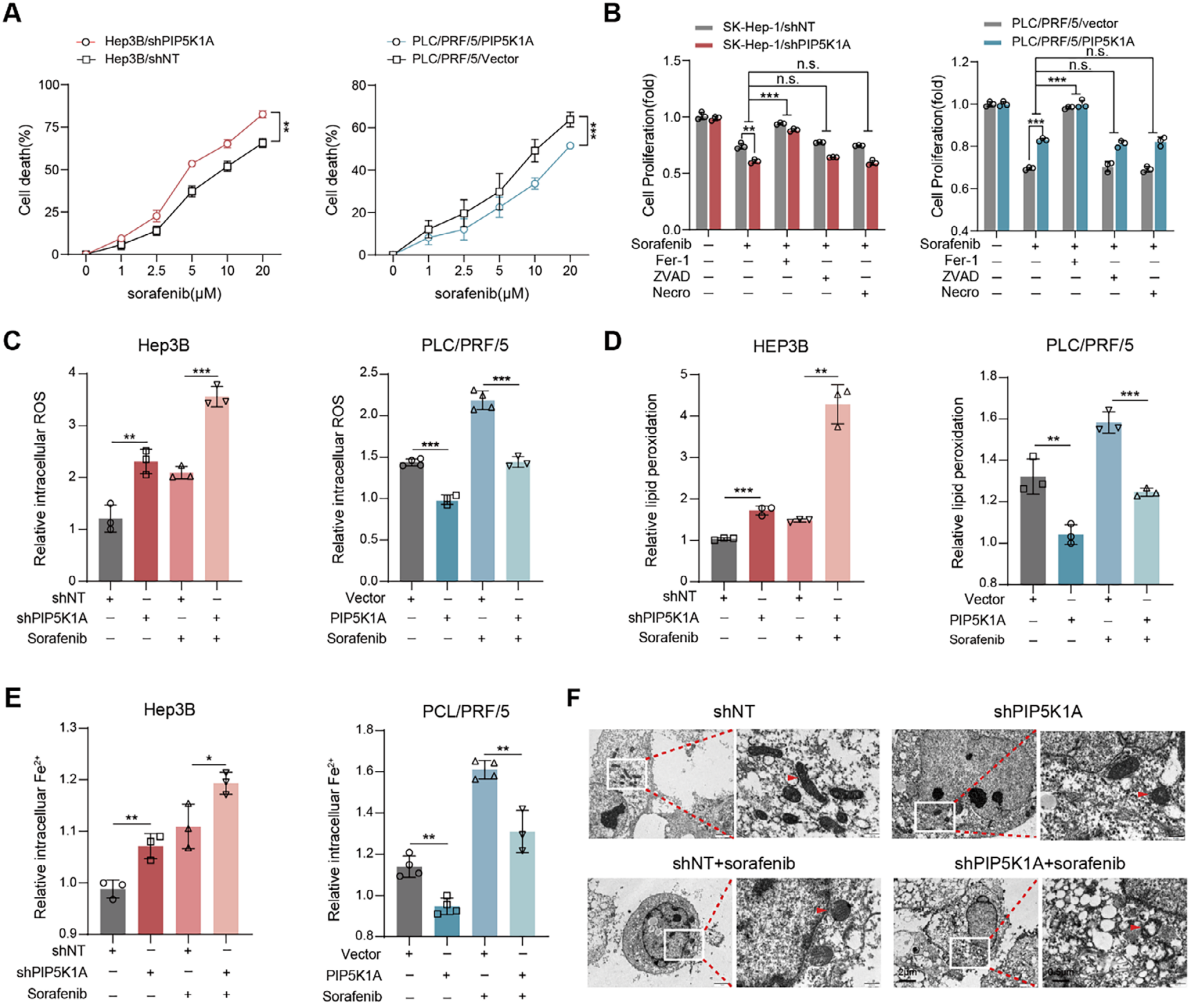
PIP5K1A protects HCC cells from sorafenib‐induced death by inhibiting ferroptosis. A) Hep3B/shPIP5K1A and PLC/PRF/5/PIP5K1A cells and their negative controls were treated with increasing concentrations of sorafenib (0, 1, 2.5, 5, 10 and 20 × 10^−6^ m) for 24 h, and the viability of cells was measured using CCK8 assay (*n* = 3). B) Hep3B/shPIP5K1A and PLC/PRF/5/PIP5K1A cells and their negative controls were treated with or without sorafenib (5 µm) for 24 h in the presence of different cell death inhibitors (Fer‐1: 10 µm; ZVAD: 10 µm; Necro: 1 µm). Cell viability was determined using CCK8 assay (*n* = 3). C–E) Indicated cells were treated with or without sorafenib (5 µm) for 24 h, then collected and used to detect total ROS, lipid ROS and Fe^2+^ levels by flow cytometry (*n* = 3). F) TEM was conducted in Hep3B/shPIP5K1A and control groups after treatment with either control or sorafenib (5 µm) for 24 h. The red arrows showed the shrunken mitochondria. Data are presented as the means ± SD. *P < 0.05; **P < 0.01; ***P < 0.001; n.s., not significant. Abbreviations: HCC: hepatocellular carcinoma; ROS: reactive oxygen species; CCK8: Cell Counting Kit‐8; Fer‐1: Ferrostatin‐1; ZVAD: ZVAD‐FMK; Necro: Necrosulfonamide.

Given the sorafenib is a recognized inducer of ferroptosis in HCC, we sought to determine if PIP5K1A could be a potential target for sorafenib‐induced ferroptosis. Notably, PIP5K1A knockdown enhanced sorafenib‐induced cell death, whereas PIP5K1A overexpression attenuated this effect (**Figure** **4**A). To clarify the form of cell death induced by PIP5K1A modulation under sorafenib treatment, we assessed the viability of HCC cells in the presence of a ferroptosis inhibitor (Fer‐1), an apoptosis inhibitor (ZVAD‐FMK), and a necroptosis inhibitor (Necro). As shown in Figure 4B, sorafenib‐induced growth inhibition in all tested cell lines (PLC/PRF/5‐PIP5K1A, SK‐Hep‐1‐shPIP5K1A, and their respective controls) was specifically rescued by Fer‐1, but not by apoptosis or necroptosis inhibitors. Ferroptosis is characterized by lipid peroxidation accumulation, ferrous iron overload, and mitochondrial alterations including reduced mitochondrial volume, condensed mitochondrial membranes, and decreased or absent mitochondrial cristae. As expected, PIP5K1A knockdown significantly enhanced sorafenib‐induced ferroptosis, as evidenced by elevated levels of total ROS, lipid ROS and iron (Figure 4C–E and Figure S3A–C, Supporting Information). In contrast, PIP5K1A overexpression significantly reduced intracellular total ROS, lipid ROS, and iron levels both with and without sorafenib treatment (Figure 4C–E and Figure S3D–F, Supporting Information). Moreover, transmission electron microscope (TEM) revealed that Hep3B‐shPIP5K1A cell treated with sorafenib exhibited more pronounced mitochondrial shrinkage and higher membrane density compared to control group under sorafenib treatment (Figure 4F). Taken together, these results suggest that PIP5K1A reduces the sensitivity of HCC cells to sorafenib by inhibiting ferroptosis.

### 2.5 PIP5K1A Stabilizes the NRF2 Protein by Decreasing Its Degradation

**Figure 5:**
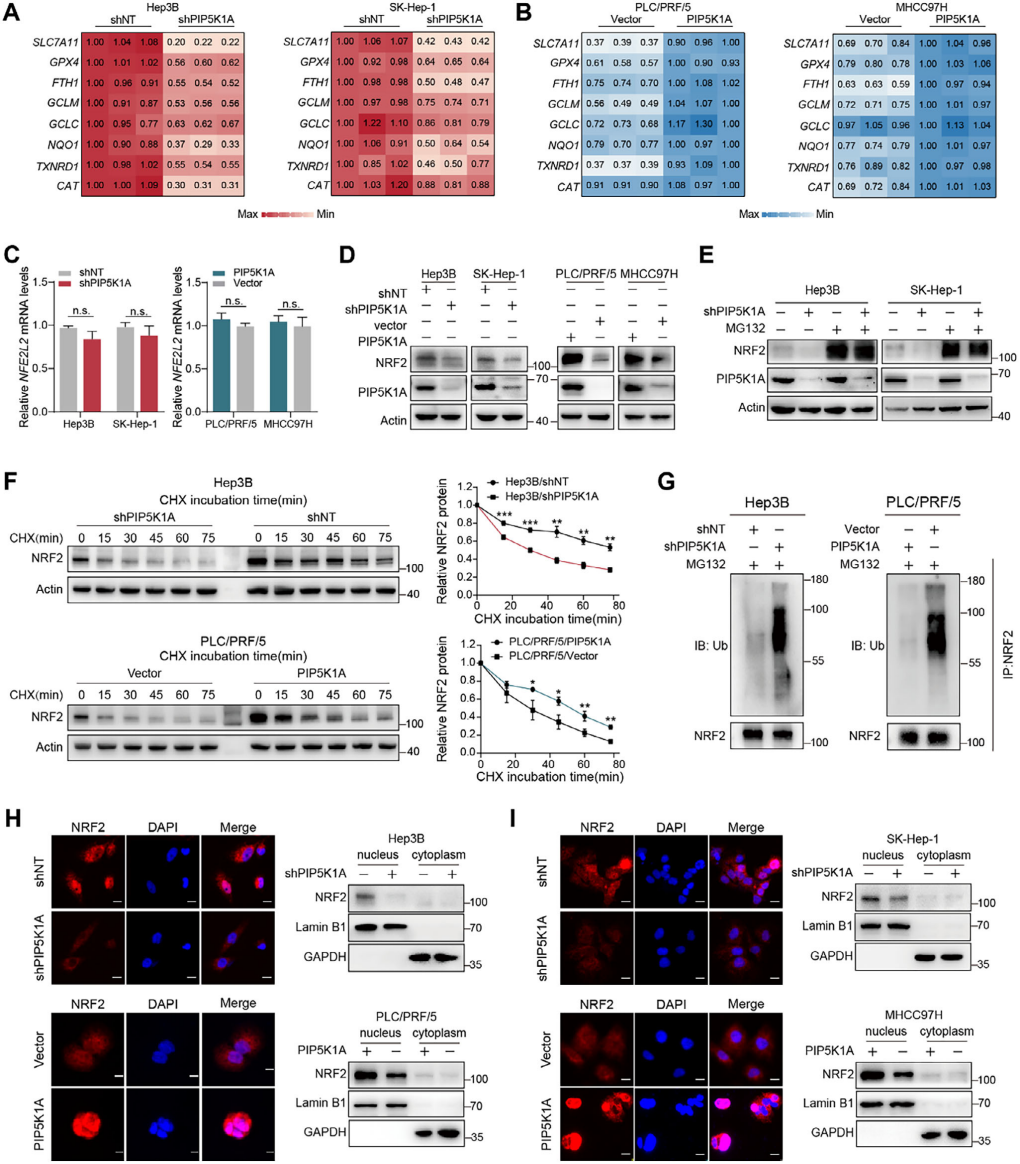
PIP5K1A stabilizes the NRF2 protein by decreasing its ubiquitin–proteasome degradation. A,B) The mRNA levels of genes involved in ferroptosis and antioxidant capacity were detected using qRT‐PCR in the indicated cells. The number shown in the heatmap represent relative mRNA expression normalized to *ACTB*. C,D) The mRNA of *NFE2L2* and the protein levels of NRF2 in the HCC cells with PIP5K1A knockdown or overexpression. E) Hep3B and SK‐Hep‐1 cells with PIP5K1A knockdown and negative control were treated with or without MG132 (10 × 10^−6^ m) for 4 h, followed by detection of NRF2 protein levels via western blotting. F) NRF2 degradation half‐life of HCC cells with PIP5K1A overexpression or knockdown were evaluated using western blotting. Cells were incubated with 20 µg mL^−1^ CHX and lysed at the indicated time points. G) NRF2 ubiquitination levels of HCC cells with PIP5K1A overexpression or knockdown. The cell lysates from indicated cells treated with MG132 (10 µm) for 6 h were immunoprecipitated with anti‐NRF2, and subjected to western blotting analysis with anti‐ubiquitin antibody. H,I) PIP5K1A promoted the nuclear translocation of NRF2 in HCC cells. Immunofluorescence was used to observe the location of NRF2 in the indicated cells. Red: NRF2; Blue: DAPI. Scale bar: 10 µm. Protein levels of NRF2 in the cytoplasm and nucleus from the indicated cells were determined using western blotting. Data are presented as the mean ± SD (*n* = 3). *P < 0.05; **P < 0.01; ***P < 0.001; n.s., not significant. Abbreviations: HCC: hepatocellular carcinoma; CHX: cycloheximide; qRT‐PCR: quantitative real‐time polymerase chain reaction.

To explore how PIP5K1A regulates redox homeostasis and ferroptosis, we examined the expression of antioxidant and ferroptosis‐related genes in cells with either PIP5K1A overexpression or knockdown. PIP5K1A knockdown decreases mRNA levels of these genes in Hep3B and SK‐Hep‐1 cells, whereas overexpression significantly increased their expression in PLC/PRF/5 and MHCC97H cells (**Figure** **5**A,B). Since NRF2 is a recognized key regulator of ferroptosis and antioxidant response, we investigated whether PIP5K1A modulates NRF2 signaling. As shown in Figure 5C,D and Figure S4A (Supporting Information), PIP5K1A overexpression and knockdown had no effects on *NFE2L2* mRNA levels but upregulated and downregulated NRF2 protein levels, respectively, suggesting that PIP5K1A may regulate NRF2 stability at the post‐transcriptional stage. This was confirmed by MG132 (proteasome inhibitor) treatment, which rescued NRF2 protein levels in PIP5K1A‐knockdown cells (Figure 5E). Next, we investigated the degradation rate of NRF2 and found that the NRF2 protein degraded more rapidly in the PIP5K1A‐knockdown group but was more stable in the PIP5K1A‐overexpressing group (Figure 5F). Consistently, PIP5K1A knockdown markedly increased NRF2 ubiquitination, whereas PIP5K1A overexpression significantly reduced it (Figure 5G). We then analyzed the effect of PIP5K1A on the nuclear translocation of NRF2. Both western blotting and immunofluorescence assays showed that nuclear NRF2 protein levels were markedly downregulated by PIP5K1A knockdown and upregulated by PIP5K1A overexpression (Figure 5H,I).

As nuclear translocation of NRF2 can be PI3K/AKT‐dependent, we investigated whether the PIP5K1A‐mediated activation of NRF2 depends on the PI3K/AKT pathway. To test this hypothesis, we assessed NRF2 expression in MHCC97H‐PIP5K1A cells after treatment with MK‐2206, the AKT inhibitor, for 24 h. Western blotting showed that MK‐2206 did not significantly alter NRF2 protein levels (Figure S4B, Supporting Information). These findings suggest that PIP5K1A inhibits ferroptosis by decreasing the ubiquitin‐proteasome degradation of NRF2 in HCC cells, independent of the PI3K/AKT pathway.

### 2.6 PIP5K1A Competes with NRF2 for KEAP1 Binding

**Figure 6:**
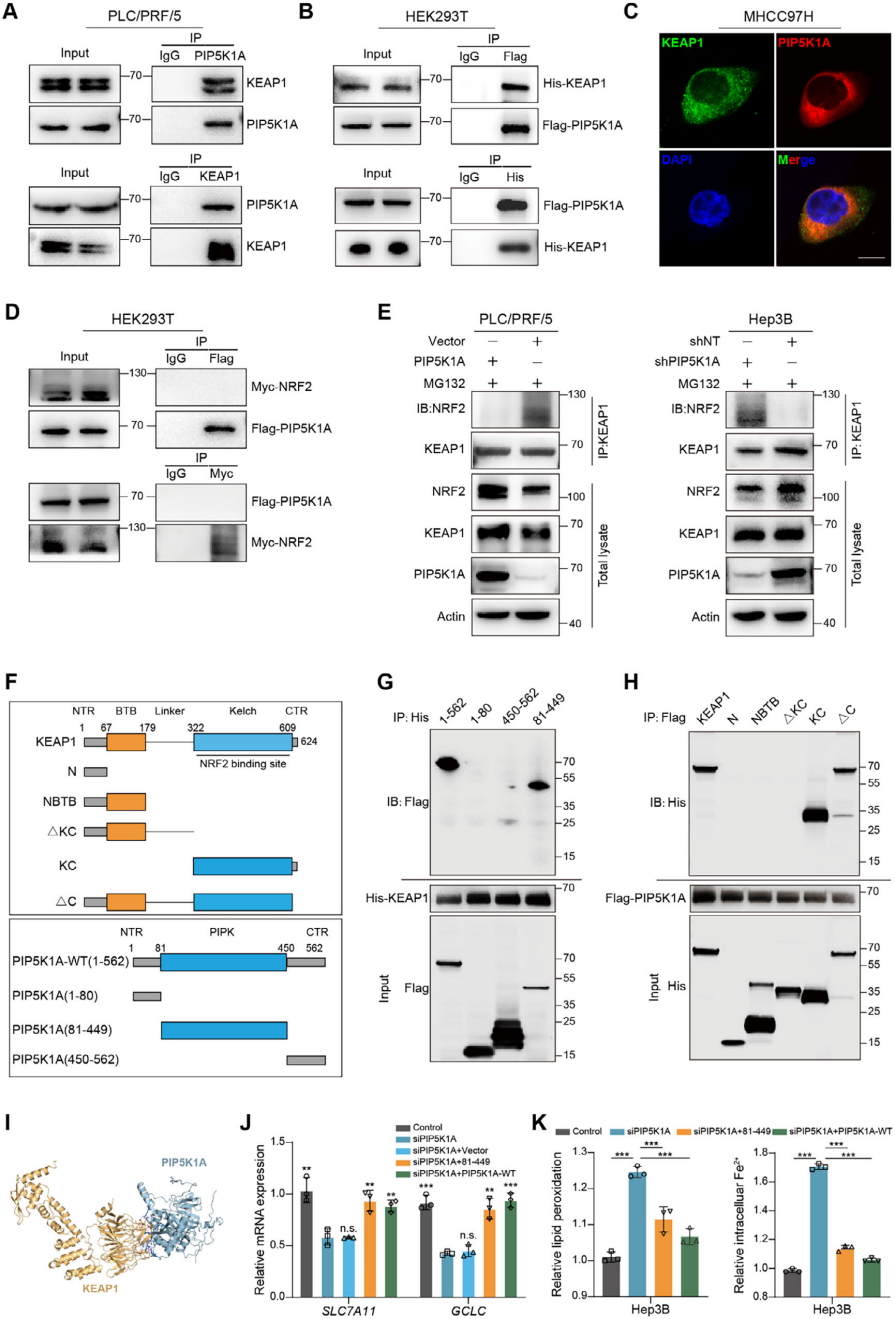
PIP5K1A competes with NRF2 for KEAP1 binding in HCC cells. A) Co‐IP assay of endogenous KEAP1 and PIP5K1A interaction in PLC/PRF/5 cells; IgG was used as control. B) HEK293T cells were co‐transfected with Flag‐PIP5K1A and His‐KEAP1 plasmids, and exogenous KEAP1‐PIP5K1A binding was analyzed by Co‐IP. C) The localization of endogenous PIP5K1A and KEAP1 in MHCC97H cell was determined by immunofluorescence assay and visualized by confocal microscopy. Scale bar: 10 µm. D) HEK293T cells were co‐transfected with Flag‐PIP5K1A and Myc‐NRF2 plasmids, and exogenous NRF2‐PIP5K1A binding was analyzed by Co‐IP. E) PIP5K1A modulates KEAP1‐NRF2 affinity. PIP5K1A‐knockdown or ‐overexpressing HCC cells and controls were treated with MG132 (10 µm) for 6 h, then Co‐IP with KEAP1 antibody followed by western blotting with NRF2 antibody. F) Schematic depiction of wild‐type and deletion mutants of His‐tagged KEAP1 and Flag‐tagged PIP5K1A. G) The interaction of exogenous His‐KEAP1‐WT with FLAG‐PIP5K1A‐WT or truncated mutants of PIP5K1A in HEK293T cells. H) The interaction of exogenous FLAG‐PIP5K1A‐WT with His‐KEAP1‐WT or truncated mutants of KEAP1 in HEK293T cells. I) The molecular model of PIP5K1A and KEAP1 interaction simulated by HDOCK server. J) PIP5K1A‐knockdown Hep3B cells were transfected with vector, truncated PIP5K1A (81–449) or Flag‐PIP5K1A. The mRNA levels of *SLC7A11* and *GCLC* were analyzed using qRT‐PCR (*n* = 3). K) Flow cytometry analysis of lipid ROS and iron levels in PIP5K1A‐knockdown Hep3B cells rescued with PIP5K1A (81–449) or Flag‐PIP5K1A (*n* = 3). Data are presented as the mean ± SD. *P < 0.05; **P < 0.01; ***P < 0.001; n.s., not significant. Abbreviations: HCC: hepatocellular carcinoma; Co‐IP: Co‐immunoprecipitation; ROS: reactive oxygen species.

Given that KEAP1 is a well‐known regulator of NRF2 ubiquitination and degradation, we speculated that PIP5K1A‐induced upregulation of NRF2 might dependent on KEAP1. We first measured the effects of PIP5K1A knockdown or overexpression on KEAP1 expression, and found that PIP5K1A did not affect KEAP1 protein levels (Figure S5A, Supporting Information). Therefore, we explored whether PIP5K1A interacts with KEAP1 to prevent the degradation of NRF2. Co‐immunoprecipitation (Co‐IP) assays showed that endogenous PIP5K1A could bind to endogenous KEAP1 (**Figure** **6**A), which was confirmed using exogenous Flag‐PIP5K1A and His‐KEAP1 in HEK293T cells (Figure 6B). Moreover, confocal microscopy and immunofluorescence staining confirmed that PIP5K1A and KEAP1 were predominantly colocalized in the cytoplasm of MHCC97H cells (Figure 6C). However, the Co‐IP assay showed that Flag‐PIP5K1A did not interact with Myc‐NRF2 in 293T cells (Figure 6D). Next, we explored the effects of PIP5K1A knockdown or overexpression on the binding of NRF2 to KEAP1. As shown in Figure 6E, KEAP1 bound more to NRF2 after PIP5K1A knockdown, whereas the binding of KEAP1 to NRF2 was reduced after PIP5K1A overexpression. It has been reported that NRF2 regulates cellular redox homeostasis through positive‐feedback loop with other proteins.^[^ ^15^, ^16^ ^]^ To determine whether NRF2 modulates PIP5K1A expression, we assessed whether NRF2 activation influences PIP5K1A expression. Treatment of Hep3B cells with the NRF2 activators tBHQ and SFN successfully upregulated NRF2 levels but did not alter PIP5K1A protein expression (Figure S5B, Supporting Information), demonstrating that PIP5K1A is not regulated by NRF2.

To identify the KEAP1 domain responsible for PIP5K1A binding, we constructed a series of KEAP1 truncated plasmids according to previous studies^[^ ^16^ ^]^ (Figure 6F). These constructs were co‐transfected with full‐length Flag‐tagged PIP5K1A into HEK293T cells. Co‐IP assays showed that Flag‐PIP5K1A specifically interacted with full length and other two truncated plasmids (N1‐609 and N322‐624) (Figure 6H), suggesting that only fragments containing the Kelch domain could interact with PIP5K1A. Next, to map the domain of PIP5K1A required for KEAP1 binding, we constructed three truncated plasmids expressing amino acids 1–80 (N‐terminal tail domain), 81–449 (phosphatidylinositol‐phosphate kinase domain (PIPK domain)), and 450–562 (C‐terminal tail domain) of PIP5K1A (Figure 6F). As shown in Figure 6G, the PIPK domain‐containing fragment (N81–449) interacts with KEAP1. To determine whether PIP5K1A binds directly to KEAP1, PyMOL and HDOCK software were used to predict the molecular docking structure of PIP5K1A and KEAP1. The docking score of PIP5K1A to KEAP1 shown by 3D structure (Figure 6I) was −292.82, with a confidence score of 0.9456. The specific residues involved in the protein–protein interactions are shown in Figure S6 (Supporting Information). Next, we investigated the role of the PIPK domain in regulating ferroptosis. As shown in Figure 6J,K, the PIP5K1A fragment N81–449 was able to restore the expression of NRF2 target genes and reversed the increased levels of lipid ROS and intracellular Fe^2+^ caused by PIP5K1A knockdown. Taken together, these results reveal that PIP5K1A competes with NRF2 for KEAP1 binding, thereby inhibiting the stability of the KEAP1–NRF2 complex and subsequent NRF2 degradation.

### 2.7 PIP5K1A Drives Growth and Suppression of Sorafenib‐Induced Ferroptosis of HCC Cells via Regulation of NRF2

**Figure 7:**
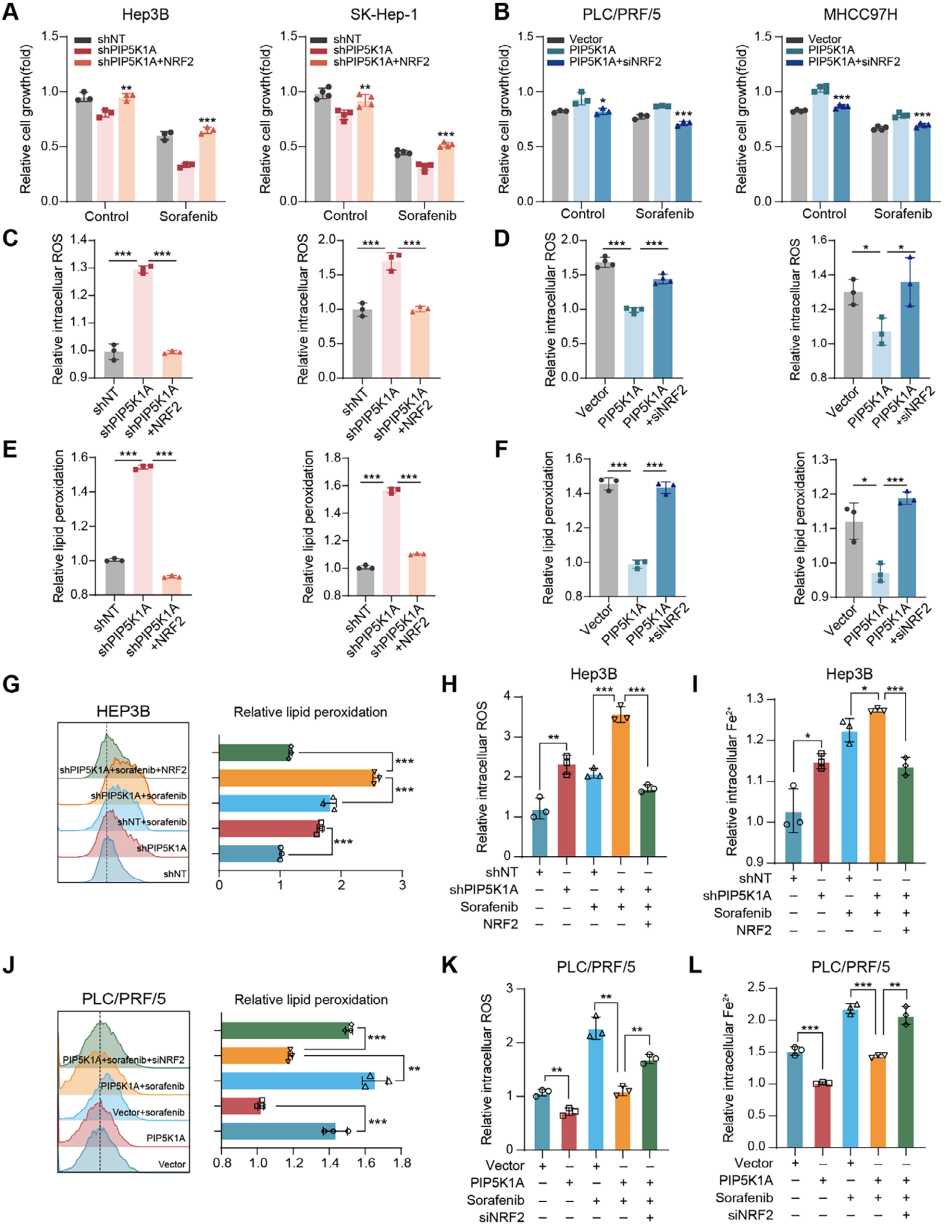
PIP5K1A inhibits sorafenib‐induced ferroptosis by activating NRF2 in HCC cells. A,B) HCC cells with PIP5K1A knockdown or overexpression were transfected with NRF2 plasmids or NRF2‐siRNA, respectively, followed by treatment with or without sorafenib (5 µm) for 24 h. Cell viability was detected using CCK8 assay. C–F) Total and lipid ROS levels were assessed by flow cytometry in PIP5K1A‐knockdown or overexpressing HCC cells transfected with NRF2 plasmids or NRF2‐siRNA, respectively. G–I) The control and PIP5K1A‐knockdown HCC cells transfected with or without NRF2 plasmids, were treated with DMSO or sorafenib (5 × 10^−6^ m) for 24 h. Lipid ROS, total ROS, and Fe^2^⁺ levels were assessed by flow cytometry. J–L) The vector and PIP5K1A‐overpressing cells transfected with or without NRF2‐siRNA, were treated with DMSO or sorafenib (5 × 10^−6^ m) for 24 h. Lipid ROS, total ROS, and Fe^2^⁺ levels were measured by flow cytometry. All data are presented as the means ± SD (*n* = 3). *P < 0.05, **P < 0.01, ***P < 0.001. Abbreviations: HCC: hepatocellular carcinoma; ROS: reactive oxygen species; CCK8: Cell Counting Kit‐8.

To further explore the role of NRF2 in PIP5K1A‐induced growth and sorafenib resistance of HCC cells, we altered NRF2 expression by transfecting PIP5K1A‐overexpressing cells with NRF2‐siRNA and PIP5K1A‐knockdown cells with NRF2‐overexpression plasmid. Western blotting was used to confirm the transfection efficiency (Figure S7A,B, Supporting Information). Overexpression of NRF2 enhanced cell growth (**Figure** **7**A) and blocked the increase in total and lipid ROS levels (Figure 7C,E) in PIP5K1A‐knockdown HCC cells. In contrast, NRF2 inhibition reduced cell growth (Figure 7B) and reversed the decrease in total and lipid ROS levels (Figure 7D,F) in PIP5K1A‐overexpressing cells. To investigate whether PIP5K1A mediated sorafenib‐induced ferroptosis via NRF2 regulation, we conducted the aforementioned experiments in the context of sorafenib treatment. As shown in Figure 7A,B, overexpression of NRF2 rescued the growth inhibition of PIP5K1A‐knockdown cells upon sorafenib treatment, whereas suppression of NRF2 enhanced the sensitivity of PIP5K1A‐overexpressing cells to sorafenib treatment. Consistent with these findings, flow cytometry analysis indicated that PIP5K1A knockdown significantly increased the levels of lipid ROS, total ROS, and Fe^2+^ compared with those in the control group under sorafenib treatment, and these effects could be reversed by NRF2 overexpression (Figure 7G–I and Figure S7C–E, Supporting Information). Conversely, PIP5K1A overexpression prevented the increase in lipid ROS, total ROS, and Fe^2+^ levels compared with those in vector control cells after sorafenib treatment, and these effects were reversed by NRF2 inhibition (Figure 7J–L and Figure S7F–H, Supporting Information). These results suggest that PIP5K1A promotes HCC cell growth and inhibits sorafenib‐induced ferroptosis in a NRF2‐dependent manner.

### 2.8 PIP5K1A Inhibitor Sensitizes the Effect of Sorafenib Treatment in HCC

**Figure 8:**
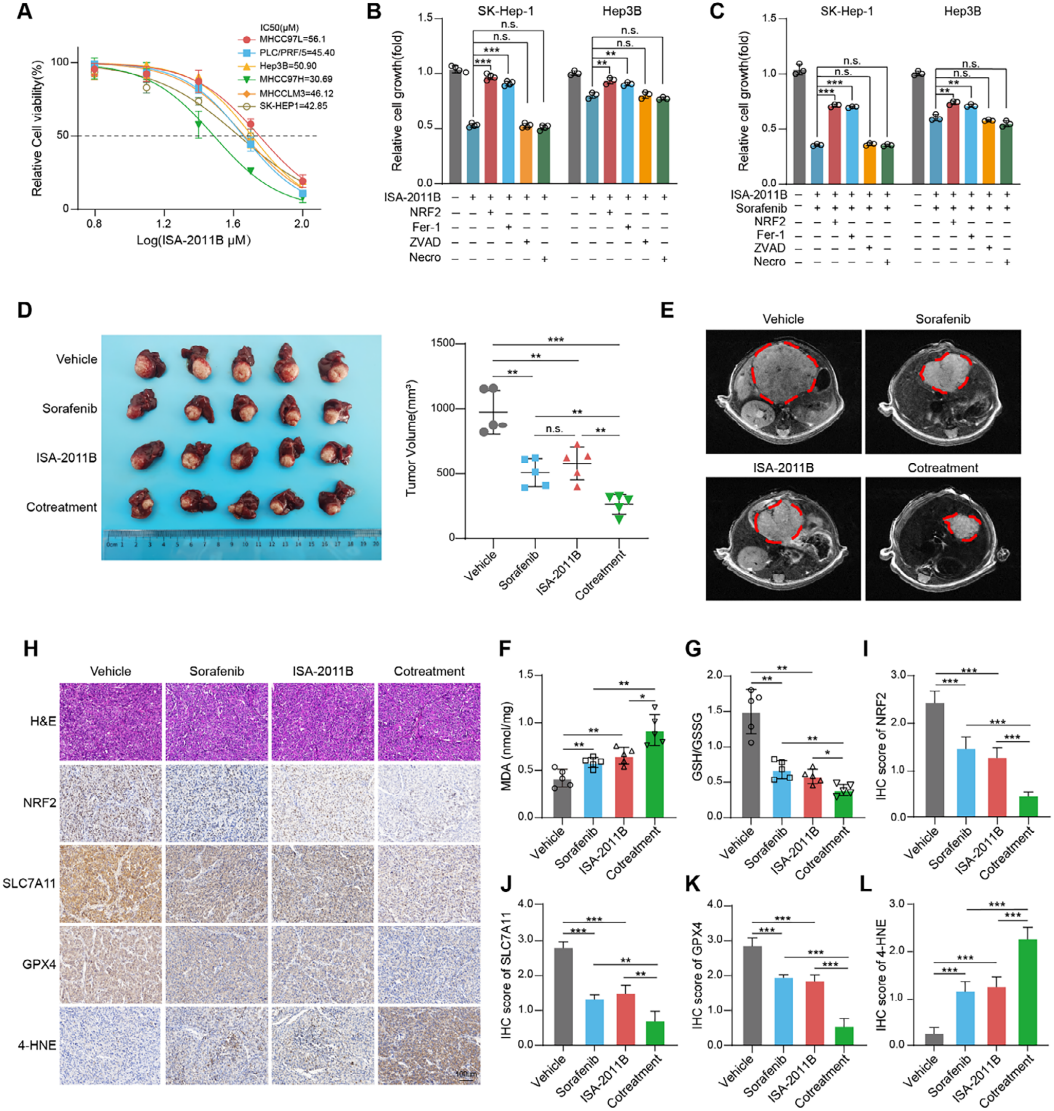
PIP5K1A inhibition sensitizes HCC cells to sorafenib treatment. A) HCC cells (PLC, Hep3B, 97L, 97H, LM3, and SK‐HEP‐1) were treated with increasing doses of ISA‐2011B (6.25, 12.5, 25, 50, and 100 µm) for 24 h, and the cell viability was measured by CCK8 assay (*n* = 3). IC50 values of each cell line are summarized in the top‐right corner. B,C) HCC cells were treated with ISA‐2011B (50 µm‐) alone or in combination with sorafenib (5 × 10^−6^ m) in the presence of cell death inhibitors (Fer‐1: 10 µm; ZVAD: 10 µm; Necro: 1 µm) or NRF2 plasmid transfection for 24 h. Cell viability was measured using CCK8 assay (*n* = 3). D) The orthotopic xenograft models derived from MHCC97H cells were treated with sorafenib, ISA‐2011B or combination therapy, and the representative images and volumes of the tumors are presented (*n* = 5). E) Representative MRI of orthotopic liver tumors form the indicated groups treated with sorafenib, ISA‐2011B or combination therapy on the 28th day are shown. F,G) MDA levels and GSH/GSSG ratios in liver tumor tissues from the indicated groups were detected (*n* = 5). H) The representative H&E staining and IHC images of NRF2, SLC7A11, GPX4, and 4‐HNE in tumor tissues of indicated groups are shown. Scale bar: 100 µm. I–L) Quantification of NRF2, SLC7A11, GPX4, and 4‐HNE protein expression in tumor tissues of indicated groups (*n* = 5). Data were presented as the mean ± SD. *P < 0.05; **P < 0.01; ***P < 0.001; n.s., not significant. Abbreviations: HCC: hepatocellular carcinoma; IHC, immunohistochemistry; MRI, magnetic resonance imaging; CCK8: Cell Counting Kit‐8; Fer‐1: Ferrostatin‐1; ZVAD: ZVAD‐FMK; Necro: Necrosulfonamide.

After confirming the biological function of PIP5K1A, we evaluated the therapeutic potential of ISA‐2011B, a selective PIP5K1A inhibitor, in inhibiting ferroptosis and sensitizing HCC cells to sorafenib. As shown in **Figure** **8**A, ISA‐2011B reduced cell viability in a dose‐dependent manner in all tested HCC cell lines. The 50% inhibitory concentration (IC50) values ranged from 30.69 (MHCC97H) to 56.1 µmol L^−1^ (MHCC97L) at 24 h. To assess the efficacy of ISA‐2011B in vivo, we established subcutaneous xenografts by inoculating SK‐Hep‐1 cells into the flanks of nude mice. Treatment with ISA‐2011B, initiated 7 d post‐inoculation, significantly reduced tumor volume and weight after two weeks with good tolerance (Figure S8A–D, Supporting Information).

Next, we examined the effects of ISA‐2011B alone or in combination with sorafenib on the viability of SK‐Hep‐1 and Hep3B cells. Notably, only ferroptosis inhibitor and NRF2 overexpression protected cells from growth inhibition induced by ISA‐2011B alone or in combination with sorafenib (Figure 8B,C). Moreover, the level of lipid ROS was higher in the combination treatment group than that in the monotherapy group (Figure S8E,F, Supporting Information). For subsequent animal studies, MHCC97H orthotopic xenograft models were employed. Consistent with in vitro results, ISA‐2011B synergized with sorafenib to achieve stronger tumor inhibition effect compared to either monotherapy (Figure 8D,E). No significant toxicity was observed in major organs (liver, lung, or kidney), nor were there changes in body weight or serum indicators of liver function across the treatment groups (Figure S8G–I, Supporting Information). These results demonstrate that the combination of ISA‐2011B and sorafenib exhibits a more potent effect in suppressing HCC proliferation compared to monotherapy, with good tolerance in mice.

To determine whether ISA‐2011B sensitizes the effect of sorafenib treatment in *vivo* by regulating ferroptosis, we measured the GSH/GSSG ratio, malondialdehyde (MDA) levels, and expression of ferroptosis‐associated genes in liver tumor tissues derived from each group. As shown in Figure 8F–L, the combination group exhibited higher levels of MDA and 4‐HNE, as well as lower levels of the GSH/GSSG ratio, NRF2, SLC7A11, and GPX4 compared to either monotherapy group. Collectively, these findings demonstrate that the PIP5K1A inhibitor synergizes with sorafenib in treating preclinical HCC models by promoting ferroptosis.

### 2.9 PIP5K1A Expression Positively Correlates with NRF2 Expression in HCC Tissues, and Their Co‐overexpression Represents a Predictor for Poor Prognosis

**Figure 9:**
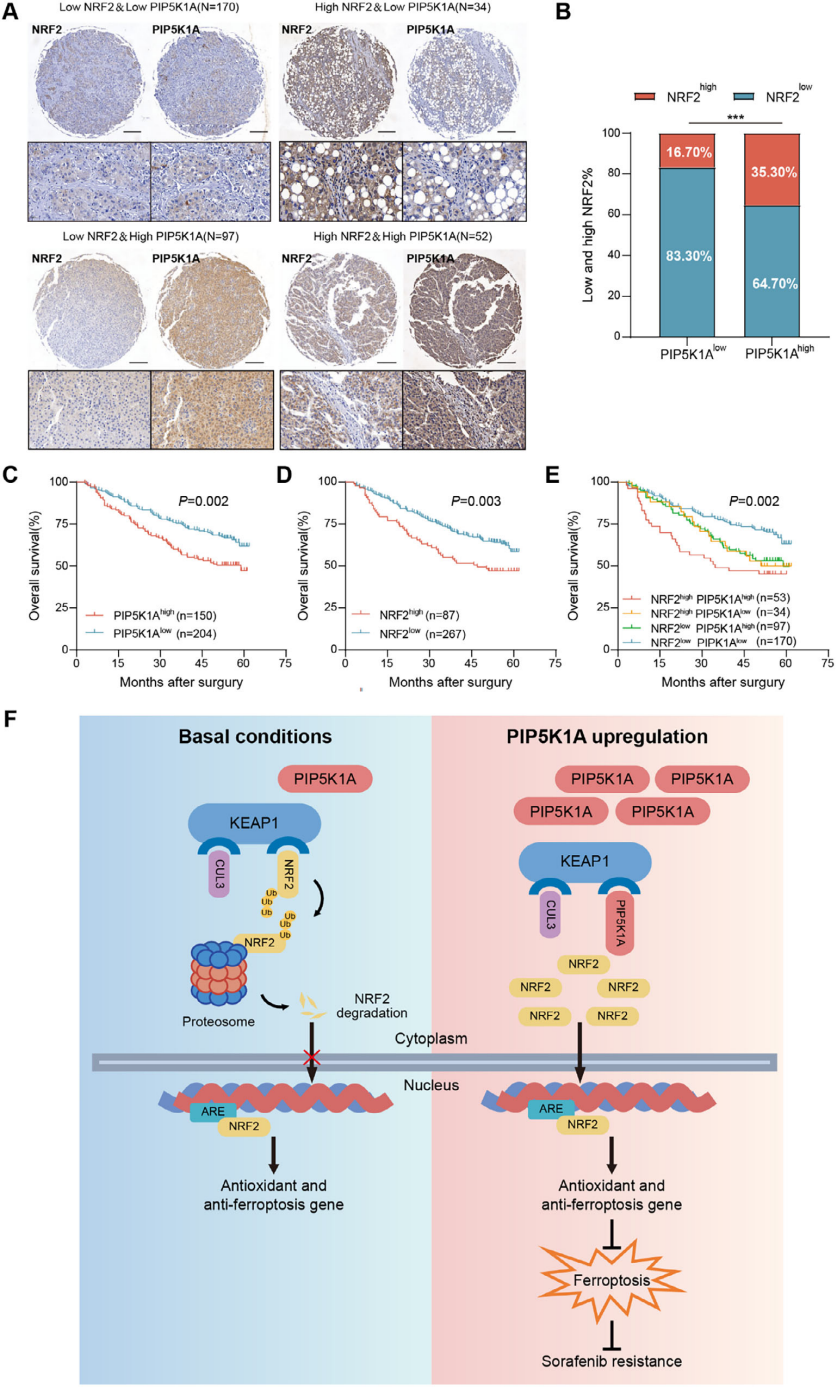
PIP5K1A expression positively correlates with NRF2 in HCC tissues, and their co‐expression predicts poor prognosis. A) Representative IHC staining of PIP5K1A and NRF2 expression patterns in the indicated groups are shown. Scale bar: 200 µm. B) Statistical analysis of the correlation between PIP5K1A and NRF2 expression patterns using Pearson χ^2^ test in 354 HCC samples. C–E) Kaplan–Meier survival analysis of OS according to the expression of PIP5K1A, NRF2, or the combined group in HCC patients using log‐rank test. F) A schematic model of PIP5K1A protects HCC against ferroptosis by sustaining NRF2 signaling activation. Upregulated PIP5K1A competes with NRF2 to bind KEAP1, which decreases the ubiquitination degradation of NRF2 and promotes its nuclear translocation, thereby inhibiting the ferroptosis signaling pathway, and ultimately promoting the growth and sorafenib‐resistance of HCC. ***P < 0.001. Abbreviations: HCC: hepatocellular carcinoma; IHC: immunohistochemistry; OS: overall survival.

To investigate the correlation between PIP5K1A and NRF2 in HCC, we examined the protein levels of PIP5K1A and NRF2 in paired tumor and adjacent normal tissues from patients with HCC in Zhongshan cohort using IHC. Based on IHC scoring, HCC patients were stratified into the following four groups as indicated in **Figure** **9A**: group I (*n* = 170), low NRF2 and low PIP5K1A; group II (*n* = 34), high NRF2 and low PIP5K1A; group III (*n* = 97), low NRF2 and high PIP5K1A; and group IV (*n* = 53), high NRF2 and high PIP5K1A. As shown in Figure 9B, tumors with high PIP5K1A expression exhibited significantly higher NRF2 staining intensity than those with low PIP5K1A. Moreover, the transcriptional level of *PIP5K1A* positively correlated with NRF2 target genes in the TCGA HCC cohort (Figure S9, Supporting Information). These results strongly indicate a significant positive correlation between PIP5K1A and NRF2 in HCC.

Finally, Kaplan–Meier survival analysis (Figure 9C) showed that patients with high PIP5K1A levels in tumor tissues had significantly shorter OS than those with low PIP5K1A levels, which is consistent with our previous results shown in Figure 1F. Similarly, patients with high NRF2 levels in the tumor tissue exhibited a significantly shorter OS than those with low NRF2 levels (Figure 9D). More importantly, patients in group IV (high NRF2/high PIP5K1A IHC scores) had the shortest OS, while those in group I (low NRF2/low PIP5K1A IHC scores) showed the longest OS among all four groups (Figure 9E). Collectively, these results demonstrate that co‐overexpression of PIP5K1A and NRF2 may serve as a robust prognostic marker for HCC.

## 3 Discussion

In the present study, we demonstrate that PIP5K1A is overexpressed in HCC and correlates with poor prognosis. Importantly, we uncover a previously unrecognized role of PIP5K1A: it promotes HCC tumorigenesis and confers HCC cells resistance to sorafenib by inhibiting ferroptosis. Mechanistically, PIP5K1A disrupts the binding of NRF2 and KEAP1, thereby stabilizing the NRF2 protein by reducing its ubiquitination‐mediated degradation, which in turn protects HCC cells from ferroptosis. Therapeutically, inhibition of PIP5K1A effectively suppresses HCC tumorigenesis and sensitizes HCC cells to sorafenib (Figure 9F).

PIP5K1A is the primary enzyme responsible for PIP2 synthesis,^[^ ^12^ ^]^ which plays an important role in activating PI3K/AKT pathway. Numerous studies have reported that PIP5K1A is overexpressed in several malignant tumors and promotes the proliferation and migration of cancer cells.^[^ ^17^, ^18^, ^19^, ^20^, ^21^ ^]^ Recent researches have also explored PIP5K1A's role in tumors independent of the PI3K/AKT pathway. For instance, PIP5K1A has been shown to interact with a unique region of KRAS, and its depletion significantly sensitizes pancreatic cancer cell lines to inhibitors of RAS effector pathways.^[^ ^22^ ^]^ In addition, Choi et al. demonstrated that nuclear PIP5K1A stabilizes stress‐induced wild‐type and mutant p53, suggesting a potential therapeutic target for cancers with mutant p53.^[^ ^23^ ^]^ According to TCGA database, ≈16% of HCC cases harbor PIP5K1A mutations, implicating its potential oncogenic role in HCC. In this study, we reported that PIP5K1A is overexpressed in HCC and correlates with poor prognosis. Furthermore, we demonstrated that PIP5K1A drives HCC growth and sorafenib resistance by suppressing lipid peroxidation and ferrous iron accumulation. Given its strong association with tumor malignancy, elucidating the precise mechanisms by which PIP5K1A inhibits ferroptosis may provide therapeutic strategy for suppressing tumor growth and restoring sorafenib sensitivity.

It is well established that NRF2 plays a vital role in protecting cells from oxidative stress.^[^ ^24^ ^]^ The overactivation of NRF2 promotes tumor survival and drug resistance by upregulating the expression of its downstream genes, which are involved in antioxidation, cellular metabolism and iron homeostasis.^[^ ^25^ ^]^ Since sorafenib has been identified as a potential inducer of ferroptosis, the role of NRF2 in sorafenib resistance has attracted considerable attention. Wang et al. showed that Glutathione S‐transferase zeta 1 (GSTZ1), an enzyme involved in phenylalanine catabolism, promotes sorafenib‐induced ferroptosis by inhibiting the NRF2/GPX4 axis in HCC.^[^ ^26^ ^]^ Recently, Chang et al. reported that DPP9 drives tumor progression and sorafenib resistance by enhancing NRF2 stability in clear cell renal cell carcinoma.^[^ ^27^ ^]^ In a previous study, our group showed that Quiescin sulfhydryl oxidase 1 (QSOX1) suppresses the activation of epidermal growth factor receptor by promoting its ubiquitination‐medicated degradation, subsequently inhibiting NRF2 activity and facilitating sorafenib‐triggered ferroptosis in HCC.^[^ ^28^ ^]^ In addition, PIP5K1C, an isoenzyme analogous to PIP5K1A, has been reported to bind NRF2 and recruit HSP27 via its product PIP2, thereby protecting tumor cells from oxidative stress.^[^ ^29^ ^]^ These findings suggest a potential role for PIP5K1A‐mediated NRF2 regulation in sorafenib resistance. In this study, we validated the positive correlation between PIP5K1A and NRF2 levels in our cohort and found that patients with high expression of both genes had a worse prognosis. More importantly, we revealed that PIP5K1A stabilizes NRF2 and activates its downstream target genes, thereby suppressing sorafenib‐induced ferroptosis. We also demonstrate that targeting PIP5K1A with small‐molecule inhibitor is a possible therapeutic strategy to sensitize HCC cells to sorafenib.

As a key transcription factor responsible for maintaining redox homeostasis, NRF2 is regulated through finely tuned mechanisms. In the present study, we found that PIP5K1A regulates NRF2 at the protein level rather than through the mRNA expression. KEAP1 is widely recognized as a critical regulator of NRF2 protein stability. Under normal circumstances, the double‐glycine repeat (DGR, Kelch) domain of KEAP1 binds to the DLG and ETGE motifs in the NRF2‐ECH homology domain‐2 (Neh2) domain of NRF2.^[^ ^10^, ^11^ ^]^ As a substrate adaptor for the cullin 3 (CUL3)‐containing E3‐ubiquitin ligase, KEAP1 recruits CUL3 and Rbx1 to form a complex with NRF2, leading to its continuous ubiquitination and proteasomal degradation, thereby maintaining low basal NRF2 levels.^[^ ^30^ ^]^ Thus, disrupting the KEAP1‐NRF2 interaction can significantly alter NRF2 protein stability. For instance, several studies have reported that proteins containing ETGE^[^ ^31^, ^32^, ^33^ ^]^ motif or DLG^[^ ^34^ ^]^ or those resembling DLG^[^ ^35^ ^]^ or ETGE^[^ ^15^, ^16^ ^]^ motifs, compete with NRF2 for binding to the Kelch domain of KEAP1. This competition protects NRF2 from ubiquitin‐proteasome‐mediated degradation, leading to the upregulation of antioxidant enzymes. Similarly, our study demonstrated that the PIPK domain of PIP5K1A competitively binds to the Kelch domain of KEAP1, thereby altering the conformation of the KEAP1/NRF2 complex and inhibiting NRF2 degradation. In addition, NRF2 protein stability can be regulated through several KEAP1‐independent mechanisms. For instance, under normal condition, NRF2 can be phosphorylated by GSK‐3β and subsequently recognized by the β‐TrCP‐Cul1 E3 ubiquitin ligase complex, leading to its proteasome degradation^[^ ^36^ ^]^ Notably, the PI3K/AKT pathway, which is hyperactivated by PIP5K1A, has been identified as an upstream regulator of NRF2/ARE‐dependent antioxidant activity.^[^ ^37^ ^]^ However, our study demonstrated that PIP5K1A regulates NRF2 independent of the PI3K/AKT pathway, revealing a novel and direct relationship between PIP5K1A and NRF2. This regulatory mechanism is analogous to PIP5K1A's interaction with let‐7 miRNA, in which PIP5K1A interacts with XPO5 to regulate mature miRNA levels by disrupting the binding of XPO5 to pre‐let‐7 miRNAs in a kinase‐independent manner.^[^ ^38^ ^]^ Considering the importance of PIP5K1A and KEAP1/NRF2 signaling in HCC, further work is needed to fully elucidate the specific interaction sites between PIP5K1A and KEAP1.

Ferroptosis resistance is a complex process regulated by multiple molecular mechanisms. In addition to intracellular signaling pathways, such as the KEAP1/NRF2 axis, the immune microenvironment (TME) may contribute to this process by regulating immune cells. For example, itaconate, which is primarily imported from tumor‐associated macrophages via SLC13A3, can activate the NRF2‐SLC7A11 pathway, thereby enabling tumor cells to escape from ferroptosis and promoting resistance to immune checkpoint blockade.^[^ ^39^ ^]^ Therefore, further studies are warranted to investigate whether PIP5K1A regulates ferroptosis resistance through TME modulation, and if combining PIP5K1A knockdown with first‐line immunotherapy could improve therapeutic outcomes in HCC.

In conclusion, the current study reveals a novel role for PIP5K1A in suppressing ferroptosis by competitively binding to KEAP1 and subsequent stabilizing NRF2 protein, ultimately protecting HCC cells form sorafenib‐induced ferroptosis. These findings suggest that targeting PIP5K1A could be a promising approach for inhibiting HCC progression and overcoming resistance to ferroptosis inducers, such as sorafenib.

## 4 Experimental Section

### Clinical Specimens and Follow‐Up

A total of 354 patients with HCC who received curative liver resection form April 2005 to September 2008 in Zhongshan Hospital of Fudan University, were included in this study. The tumor and paired adjacent non‐tumor liver tissues were obtained from these 354 enrolled patients to construct a tissue microarray (TMA). Among these, 34 randomly selected paired frozen samples were used to measure *PIP5K1A* mRNA levels, and nine paired samples were used to assess PIP5K1A protein expression. Detailed clinicopathologic characteristics are provided in Table S1 (Supporting Information). The inclusion and exclusion criteria for patients were described in our previous study.^[^ ^28^ ^]^ OS was defined as the time between hepatectomy and death or last follow‐up. All patients were post surgically followed to June 20, 2015. The Research Ethics Committee of Zhongshan Hospital of Fudan University approved the use of human subjects involved in this study (No. 2021BAT4859), and written informed consent was obtained from each patient.

### Cell Culture and Reagents

The three human HCC cell lines (PLC/PRF/5, Hep3B, and SK‐Hep‐1) and the human embryonic kidney cell line (HEK293T) were purchased from the cell bank of the Chinese Academy of Science (Shanghai, China). The MHCC97H cell was established at the Liver Cancer Institute, Zhongshan Hospital, Fudan University. All cell lines were passaged for one month or 10 passages before a new aliquot was thawed. MHCC97H, PLC/PRF/5, Hep3B, SK‐Hep‐1, and HEK293T cells were cultured in Dulbecco's modified Eagle medium (DMEM; GNM12800‐2, GENOM) containing 10% fetal bovine serum (FBS; 10270‐106, Gibco) and 1% penicillin‐streptomycin (15140‐122, Gibco). Hep3B cell was cultured in Minimum Essential Medium (MEM; GNM41500‐2, GENOM) with 10% FBS. All cells were maintained at 37 °C in a humidified incubator (Thermo Fisher Scientific, Waltham, MA) with 5% CO_2_.

ISA‐2011B (HY‐16937), Z‐VAD(OMe)‐FMK (ZVAD; HY‐16658), Necrosulfonamide (Necro; HY‐100573), MG‐132 (HY‐13259), TBHQ (HY‐100489), and Sulforaphane (HY‐13755) were purchased from MedChemExpress (New Jersey, USA). Ferostatin‐1(Fer‐1; S7243), MK‐2206 2HCl (S1078), and Sorafenib (S7397) were purchased from Selleck Chemicals (Texas, USA).

### Plasmids and Lentiviral Transfection

The lentiviral‐based small hairpin RNA (shRNA) to silence PIP5K1A (targeting GAGCTAGTGGTTCCCTATT), PIP5K1A overexpression lentivirus, and their corresponding control lentivirus were constructed by GeneChem Co., Ltd. (Shanghai, China). All lentivirus transfections were performed according to the manufacturer's instructions, and stably infected cell lines were selected using 2 µg mL^−1^ puromycin at least two weeks. The PIP5K1A shRNA plasmid, PIP5K1A‐Flag plasmid, NRF2‐Myc‐plasmid, ubiquitin‐HA‐plasmid, KEAP1‐His‐plasmid, KEAP1‐His truncation plasmid (amino acids 1–66, 1–179, 1–321, 1–609, 322–624) and PIP5K1A‐Flag truncation plasmid (amino acids 1–80, 81–449, 450–562) were constructed and obtained from GeneChem Co., Ltd. (Shanghai, China). These plasmids were transduced into HEK293 cells using HieffTrans Universal Transfection Reagent (40808ES03, Yeasen Biotechnology Co., Ltd.). Briefly, 5 µL universal‐A reagent and 2.5 µg plasmid were diluted in 125 µL Opti‐MEM, and mixed with diluted universal‐B reagent (5 µL universal‐B reagent were diluted into 125 µL Opti‐MEM) at room temperature for 5 min. The mixture was then added to the cells for 48 h of transfection.

### Small Interfering RNA (siRNA) transfection

The siRNAs specifically targeting NRF2 and PIP5K1A were obtained from shanghai QianMo Biotechnology Co., Ltd. The siRNAs were transfected into HCC cells using QM‐RNA Transfection Kit (QMT1001) according to the manufacturer's instructions. The siRNA sequences were as follows:

si‐NRF2: 5′‐CCGGCAUUUCACUAAACACAATT‐3′;

si‐PIP5K1A: 5′‐GAGCTAGTGGTTCCCTATT‐3′;

### Detection of Total ROS, Mitochondrial ROS, Lipid Peroxidation, Intracellular Iron, and GSH/GSSG Ratio

1. Total ROS levels were measured according to the manufacturer's instructions. In brief, 5 × 10^5^ cells were incubated with 2 mL culture medium containing 5 × 10^−6^ m CM‐H2DCFDA (C6827, Invitrogen, USA) at 37 °C for 20 min without light in the plates. The cells were washed three times with PBS to remove excess CM‐H2DCFDA, harvested and resuspended in 300 µL PBS followed by flow cytometric analysis.
2. For detection of mitochondrial ROS, 5 × 10^5^ cells were incubated with 2 mL culture medium containing 5 × 10^−6^ m MitoSOX Red Mitochondrial Superoxide Indicator (M36008, Invitrogen, USA) at 37 °C for 15 min without light in the plates. Cells were washed three times with PBS, harvested and then resuspended in 300 µL PBS followed by flow cytometric analysis.
3. Lipid peroxidation assay: 5 × 10^5^ cells were incubated in 2 mL medium with 5 × 10^−6^ m of C11‐BODIPY (581/591) (#D3861, Invitrogen, USA) for 30 min at 37 °C. After washing with PBS three times to remove excess dye, the cells were harvested and suspended in 300 µL of PBS for flow cytometer analysis.
4. Iron assay: A total of 5 × 10^5^ cells were incubated in 2 mL medium with 1 × 10^−6^ m of FerroOrange (Dojindo, Japan) for 30 min at 37 °C. Subsequently, the cells were rinsed, collected and then suspended in 300 µL of PBS for flow cytometer analysis.
5. The ratio of GSH/GSSG was measured using GSH and GSSG Assay Kit (S0053, Beyotime, China) according to the manufacturer's instruction as described previously,^[^ ^40^ ^]^ and protein concentrations of each sample were used for normalization. All experiments were carried out at least in triplicate.

### Cell Proliferation and Colony Formation Assay

For Counting Kit‐8 (CCK‐8) assay, stably transfected HCC cells (either PIP5K1A‐overexpressing or PIP5K1A‐knockdown) were seeded in 96‐well plates at a density of 2000 cells/well in 100 µL complete medium. According to the protocol of CCK‐8 assay kit (Dojindo, Kumamoto, Japan), 100 µL complete medium containing 10 µL CCK‐8 reagent was added to each well at different time points (24, 48, 72, and 96 h) to replace the original medium. After the plates were incubated in dark at 37 °C for 2 h, the absorbance at 450 nm wavelength was measured to calculate cell viability.

For colony formation assays, cells were seeded in a six‐well plate (1000 cells/well) and cultured at 37 °C in a 5% CO₂ humidified atmosphere. After 14 d of culture, the cells were fixed with 4% paraformaldehyde for 15 min at room temperature and then stained with crystal violet for 30 min. The number of colonies were counted using ImageJ software (National Institute of Health, Bethesda, MD).

### IHC

The IHC procedure was performed as described previously.^[^ ^41^ ^]^ Antibodies used for IHC staining are listed in Table S2 (Supporting Information). Two independent pathologists, who were blinded to the patients’ clinical data, scored the IHC staining using a histological score (H‐score) approach. The staining intensity was graded as follows: 0 (negative), 1 (weak), 2 (intermediate), and 3 (strong). The H‐score was calculated as the staining intensity score × the percentage of target protein‐positive cells. The median H‐scores were used as cutoff to separate patients into low and high expression subgroups. IHC staining for the xenograft mouse model's tumor samples was also performed as previously described.

### RNA Extraction and Quantitative Real‐time Polymerase Chain Reaction (qRT‐PCR)

Total RNA was extracted from tissue and cell samples using TRIzol reagent (Invitrogen Life Technologies) according to the manufacturer's protocol. 1 µg of total RNA were reverse transcribed into cDNA using the RT reagent Kit with gDNA Eraser (RR047, Takara, Japan). The qRT‐PCR assay was performed using SYBR Green qPCR Master Mix (11202ES, Yeasen, China) with specific primers (Table S3, Supporting Information) on an ABI Prism 7500 Sequence Detection system (Applied Biosystems, USA), following the manufacturer's instructions. Expression data were normalized to *ACTB* reference gene level and presented as relative mRNA expression.

### Immunofluorescence and Confocal Microscopy Assay

Cells grown on coverslips were fixed with 4% formaldehyde for 15 min, permeabilized in 0.5% Triton X100 for 20 min, blocked with 5% bovine serum albumin (BSA) for 1 h at room temperature, and then incubated with primary antibodies overnight at 4 °C. On the second day, the cells were washed with PBS containing 0.1% Tween‐20 (PBST) for three times and incubated with the indicated secondary antibodies for 1 h at room temperature. Nuclei were counterstained with 4ʹ,6‐diamidino‐2‐phenylindole (DAPI) for 5 min. Fluorescent images were acquired using either a fluorescence microscope or confocal laser scanning microscope (Olympus, Tokyo, Japan). The corresponding antibodies are listed in Table S2 (Supporting Information).

### Western Blotting and Co‐IP Assay

For Western blotting, whole cell extracts were lysed in RIPA lysis buffer (Beyotime, China) containing 1% protease and phosphatase inhibitor cocktail for 20 min on ice. The nuclear and cytoplasmic proteins were extracted using NE‐PER Nuclear and Cytoplasmic Extraction Reagents (78833, Thermo Scientific, USA) according the manufacturer's instructions. The protein samples were boiled at 100 °C for 10 min in buffer, then subjected to SDS‐polyacrylamide gel electrophoresis (SDS‐PAGE) and subsequently transferred to PVDF membranes (Millipore, USA). After sequential incubation with primary and secondary antibodies, target proteins were visualized using chemiluminescence substrate.

For Co‐IP assays, the cell lysates were purified by centrifugation at 10 000× *g* for 15 min at 4 °C. The resulting supernatants were incubated overnight with indicated antibodies or IgG rotationally at 4 °C, followed by incubation with Protein A/G magnetic beads (HY‐K0202, MedChemExpress, USA) for 2 h at 4 °C rotationally. After washing four times with IP lysis buffer, the bound proteins were harvested by centrifugation and then boiled in 1 × loading buffer for 10 min at 100 °C. The interactions between proteins were performed by immunoblotting as previously described. Protein–protein interactions were analyzed by immunoblotting as previously described. Antibodies used for immunoprecipitation and Immunoblotting are listed in Table S2 (Supporting Information).

### TEM Assay

The cells were plated in 10 cm dish with a density of 6 × 10^5^ cells per dish and incubated overnight. The following day, cells were treated with vehicle control or sorafenib (5 µm). After 24 h of treatment, cells were collected and fixed with 2.5% glutaraldehyde at 4 °C, followed by treatment with a 1% osmium tetraoxide for 2 h at room temperature. Subsequently, the samples were dehydrated in gradual ethanol and embedded in epoxy resin. Representative images were captured by TEM (Tecnai G2 F20 S‐TWIN, FEI, USA) after slicing and staining with uranyl acetate and lead citrate.

### Bioinformatics

The sequencing data (TPM format) and corresponding clinical information for HCC patients were obtained from The Cancer Genome Atlas (TCGA) database (https://portal.gdc.cancer.gov/). Gene expression data were normalized using a log2^(TPM+1)^ transformation. For survival analysis, patients with a survival time of 0 and duplicate sequencing samples from the same individual were excluded, and a total of 365 patients were classified into high *PIP5K1A* group and low *PIP5K1A* group according to the optimal cutoff value which were determined using X‐tile software (Yale University, New Haven, CT). In addition, GSEA was conducted by GSEA V4.3.2 software for evaluating signaling pathways differences between high and low risk groups based on median *PIP5K1A* expression. The threshold for enriched items and pathways was |NES| > 1, NOM *p*‐value < 0.05, and *q*‐value < 0.25.

### Molecular Docking Assay

The molecular structures of PIP5K1A (UniProt ID: Q99755) and KEAP1 (UniProt ID: Q14145) were obtained from the Protein Data Bank (PDB) database. To predict the binding site between PIP5K1A and KEAP1, molecular docking was performed using HDOCK Server (https://hdock.phys.hust.edu.cn). The most optimal binding model determined by the docking score was visualized, analyzed, and mapped using PyMOL program version 2.4.0 (https://www.schrodinger.com/pymol). For protein–protein interactions, docking scores are generally considered significant when lower than −200. In addition, binding confidence was categorized as follows: high (confidence score > 0.7), moderate (0.5–0.7), or low (score < 0.5).

### RNA Sequencing

Total RNA was extracted from Hep3B‐shNT and Hep3B‐shPIP5K1A cells.

Following quality inspection, cDNA synthesis and PCR amplification were performed, and subsequent sequencing was conducted by Majorbio Technology Inc. (Shanghai, China). DEGs were identified using DESeq2 with the screening threshold of |Fold change| >1.2 and FDR < 0.05. The DEGs were then analyzed using the KEGG pathway database.

### Animal Studies

Six‐week‐old male BALB/c nude mice were purchased from Lingchang Biotechnology Co., Ltd. (Shanghai, China). To establish the subcutaneous mouse model, 1 × 10^7^ SK‐Hep‐1 shNT or SK‐Hep‐1 shPIP5K1A cells suspended in 200 µL of PBS were injected subcutaneously to the right flanks of the mice (6/group). Body weight was monitored once every other day, and the subcutaneous tumor volume was measured twice a week. After four weeks of implantation, the mice were euthanized and the tumors were dissected. The tumor volume (*V*) was calculated as *V* (mm^3^) = 0.5 × length (mm) × width (mm^2^).

For orthotopic implantation, 5 × 10^6^ MHCC97H/PIP5K1A or MHCC97H/vector cells were subcutaneously injected into the right flank of six‐week‐old male BALB/c nude mice. Fourteen days after implantation, tumors were excised and sectioned into 1 mm^3^ cubes under sterile conditions. These tumor fragments were then implanted into the right lobe of liver parenchyma of additional six‐week‐old male BALB/c nude mice. At the seventh day following implantation, mice carrying MHCC97H/PIP5K1A or MHCC97H/vector cells were randomly separated into four groups: (1) vehicle control, (2) 30 mg kg^−1^ sorafenib through oral gavage once every other day for three weeks, (3) 40 mg kg^−1^ ISA‐2011B through injection intraperitoneally once every other day, or (4) combination therapy. All treatments continued for three weeks. At the end of treatment, mice received magnetic resonance examination (MRI) with CG NOVILA 7.0T system (Shanghai Chenguang Medical Technologies Co., Ltd., China). After examination, the mice were sacrificed and the tumors were dissected for weight measurement, volumetric analysis (using the previously described formula), histological examination, and IHC analysis. All animal experiments were performed according to the guidelines of the National Academy of Sciences and the National Institutes of Health, with approval from the ethics committee of Zhongshan Hospital, Fudan University (No. 2019086).

### Statistical Analysis

All experiments were conducted at least three times or with a minimum sample size of three. Data are presented as the mean ± standard deviation (SD). Statistical analyses were performed with SPSS (23.0, IBM, USA) and Graphpad Prism 8.0 software. Differences between two groups were performed with Student's *t*‐test, while one‐way ANOVA followed by Tukey's test was used to compare date among multiple groups. Categorical variables were evaluated using Pearson χ^2^ test. Correlation between gene expression was determined by Pearson's correlation coefficient after normal distribution was assumed. OS was estimated using the Kaplan–Meier method and compared by log‐rank test. A two‐sided *p*‐value < 0.05 was considered statistically significant.

## Conflict of Interest

The authors declare no conflict of interest.

### Author Contributions

M.G., S.C., and J.S. contributed equally to this work. M.G.: conceptualization, data curation, formal analysis, investigation, methodology, project administration, writing – original draft, writing – review & editing, funding acquisition. S.C.: data curation, investigation, methodology, formal analysis, visualization. J.S.: conceptualization, data curation, formal analysis, validation, writing – review & editing. R.X.: investigation, methodology, formal analysis, resources. Z.Q.: data curation, investigation, methodology. J.L.: investigation, software. L.Z.: investigation, visualization. Y.F.: supervision, resources, validation. T.L.: funding acquisition, supervision. J.X.: conceptualization, supervision, project administration, resources, funding acquisition, writing – review & editing.

## Supporting information



Supporting Information

## Data Availability

The data that support the findings of this study are available from the corresponding author upon reasonable request.
