# Supplementary material for: PIP5K1A Suppresses Ferroptosis and Induces Sorafenib Resistance by Stabilizing NRF2 in Hepatocellular Carcinoma
Source: Adv Sci (Weinh). 2025 May 23;12(30):e04372. doi: 10.1002/advs.202504372 (PMC12376573; doi:10.1002/advs.202504372)
Supplement: Supplementary file 1 — Supporting Information [file ADVS-12-e04372-s001.docx]

**PIP5K1A Suppresses Ferroptosis and Induces Sorafenib Resistance by Stabilizing NRF2 in Hepatocellular Carcinoma**

*Mengzhou Guo^1^, Sinuo Chen^1^, Jialei Sun^1^, Ruchen Xu, Zhuoran Qi, Jie Li, Lianer Zhou, Yuan Fang*, Tianshu Liu*, Jinglin Xia**

M. Guo, T. Liu

Department of Medical Oncology, Zhongshan Hospital, Fudan University, 180 Fenglin Road, Shanghai 200032, China

E-mail: [liu.tianshu@zs-hospital.sh.cn](mailto:liu.tianshu@zs-hospital.sh.cn)

M. Guo, S. Chen, J. Sun, Z. Qi, J. Li, L. Zhou, Y. Fang, J. Xia

Liver Cancer Institute & Key Laboratory of Carcinogenesis and Cancer Invasion (Ministry of Education), Zhongshan Hospital, Fudan University, 180 Fenglin Road, Shanghai 200032, China

E-mail: [xiajinglin@fudan.edu.cn](mailto:xiajinglin@fudan.edu.cn)

J. Sun, R. Xu, Z. Qi

Department of Gastroenterology and Hepatology and Shanghai Institute of Liver Diseases, Zhongshan Hospital, Fudan University, 180 Fenglin Road, Shanghai 200032, China

Y. Fang

Department of Liver Surgery, Zhongshan Hospital, Fudan University, 180 Fenglin Road, Shanghai 200032, China

E-mail: [fangyuan1029@126.com](mailto:fangyuan1029@126.com)

^1^ These authors contributed equally to this work.

Keywords: hepatocellular carcinoma, ferroptosis, PIP5KA, KEAP1, NRF2, sorafenib resistance

**
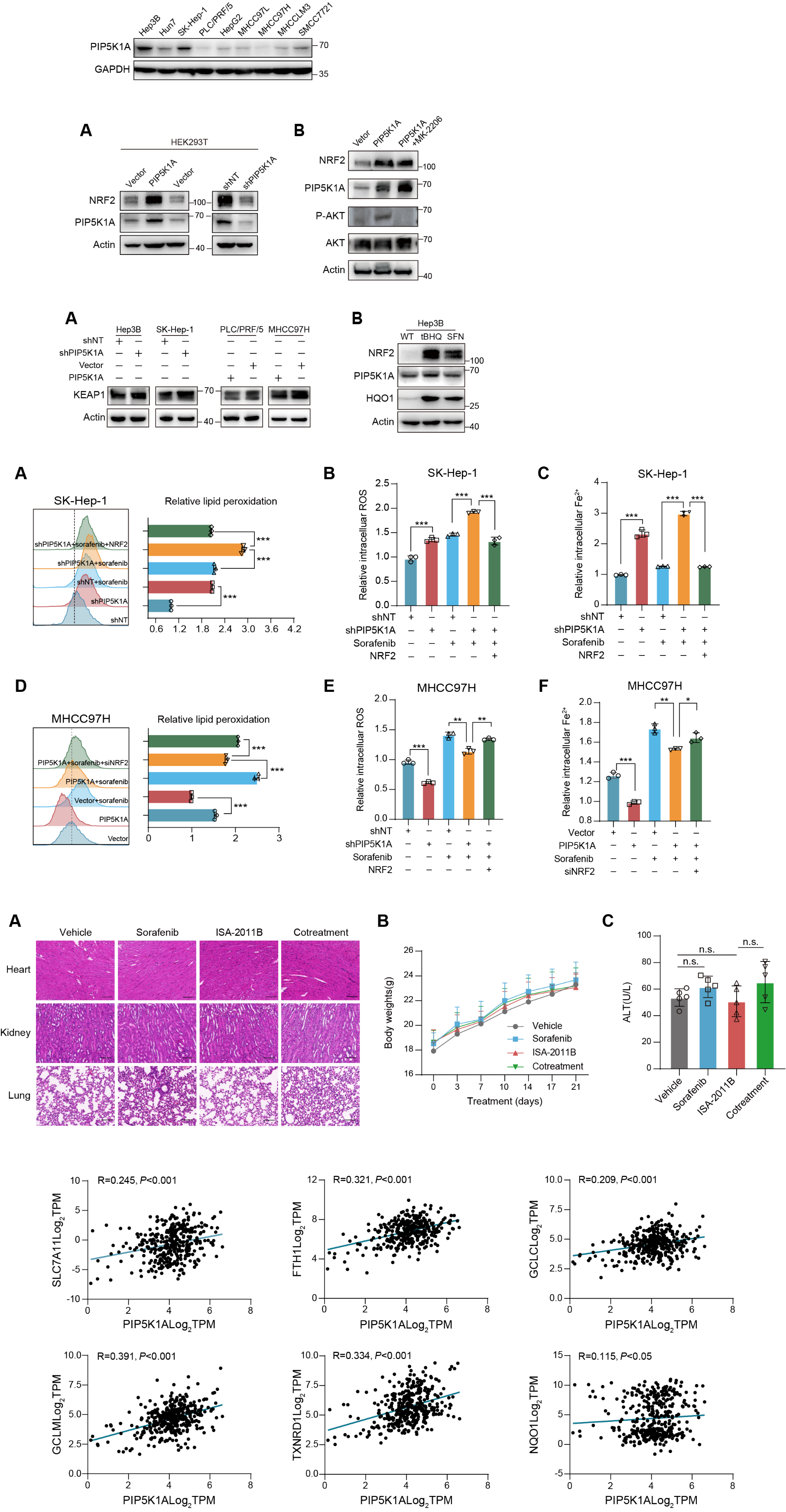
**

**Figure S1.** Protein expression of PIP5K1A in different HCC cell lines detected by western blotting.

**
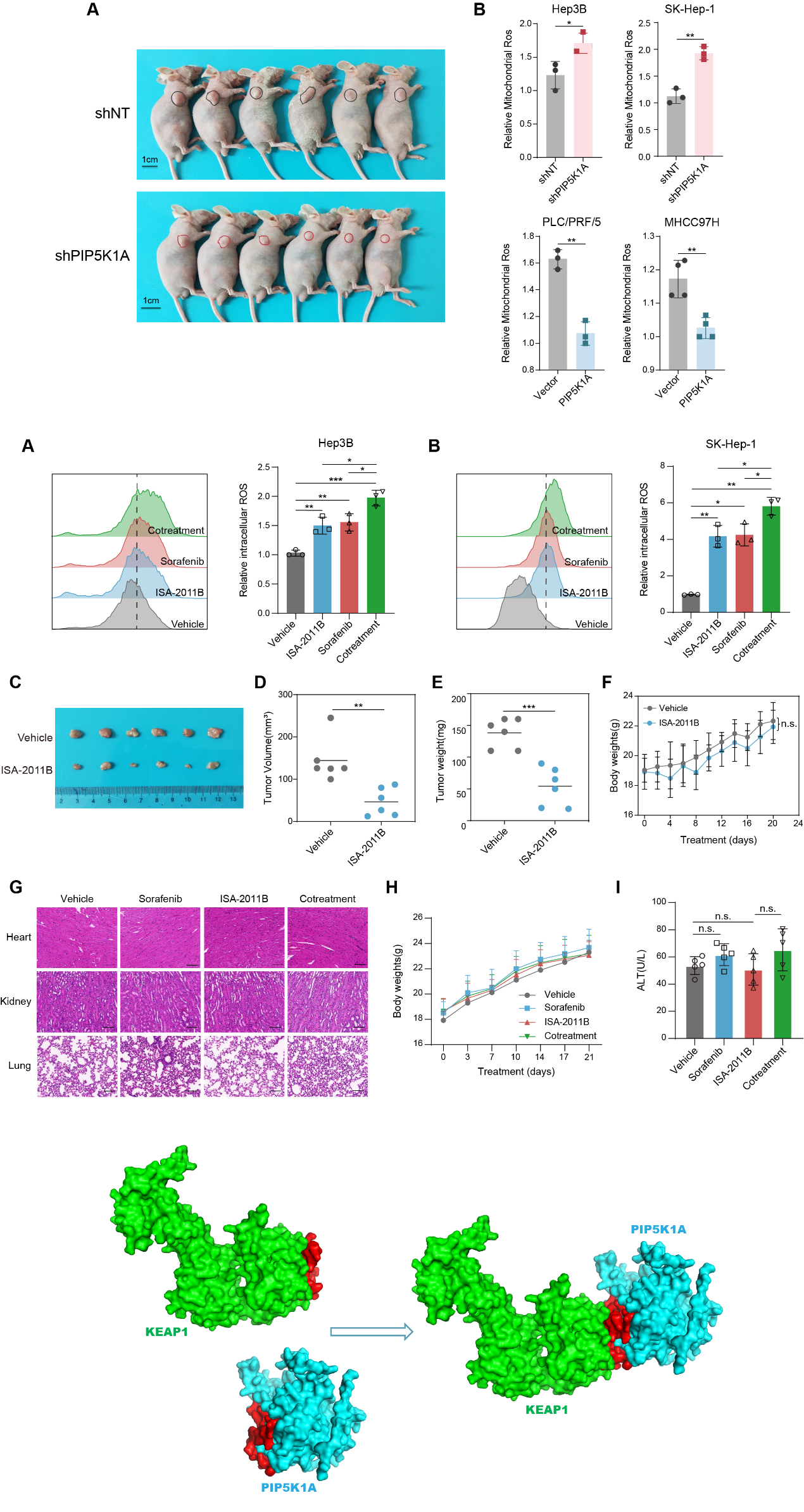
**

**Figure S2.** Knockdown of PIP5K1A inhibits HCC growth and increases mitochondria ROS level in HCC. A) Knockdown of PIP5K1A significantly inhibits HCC growth in *vivo* (n=6). B) Levels of mitochondrial ROS in the indicated cells were stained by MitoSOX Red Mitochondrial Superoxide Indicator and determined by flow cytometry (n=3). Data are presented as the mean ± SD. **P* < 0.05, ***P* < 0.01.


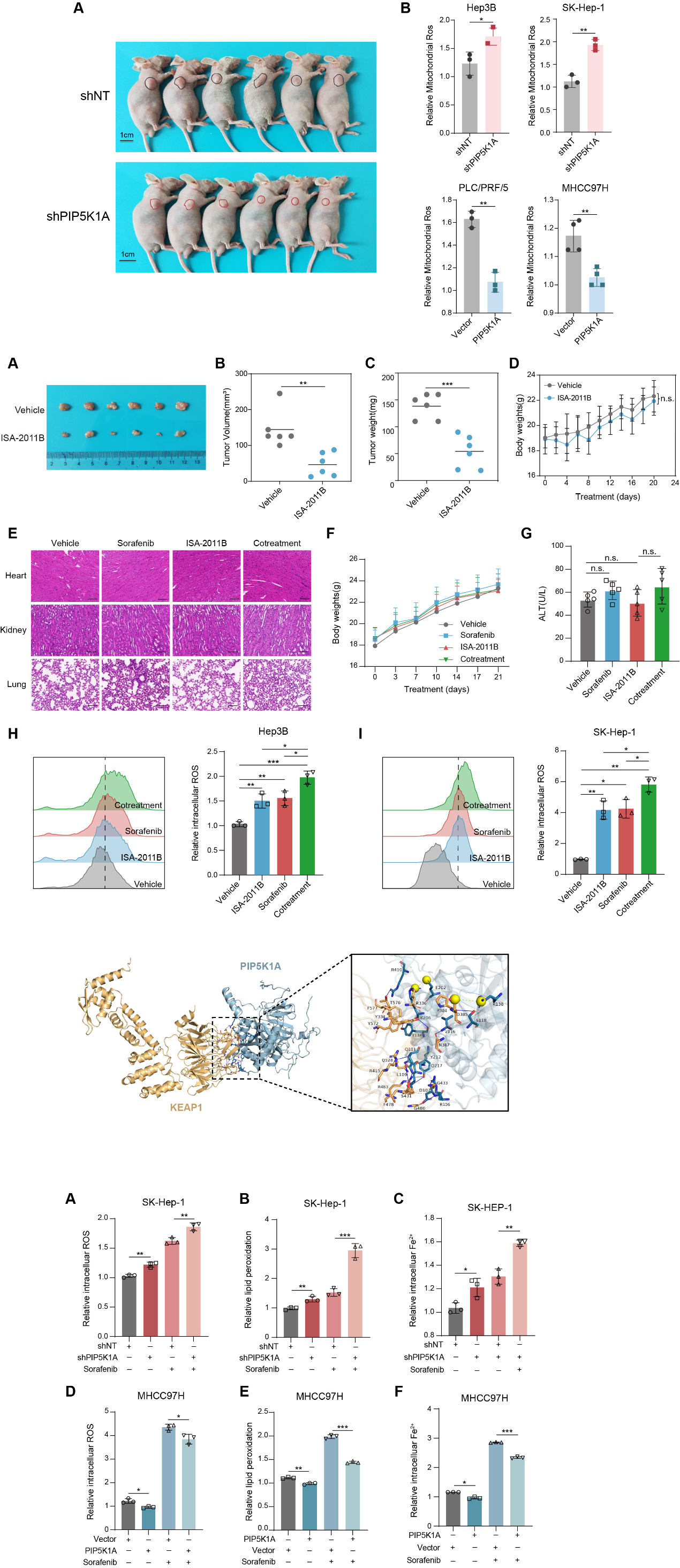


**Figure S3.** PIP5K1A suppresses sorafenib-induced ferroptosis in HCC cells. A-C) SK-Hep-1/shPIP5K1A cells and their negative control were treated with or without sorafenib (5μm) for 24 h. Cells were then collected, and total ROS, lipid ROS and Fe^2+^ levels were measured by flow cytometry. D-F) PLC/PRF/5/PIP5K1A cells and their negative control were treated with or without sorafenib (5μm) for 24 h. Cells were then collected, and total ROS, lipid ROS and Fe^2+^ levels were measured by flow cytometry. Data are presented as the mean ± SD (n=3). **P* < 0.05, ***P* < 0.01, ****P* < 0.001.

**
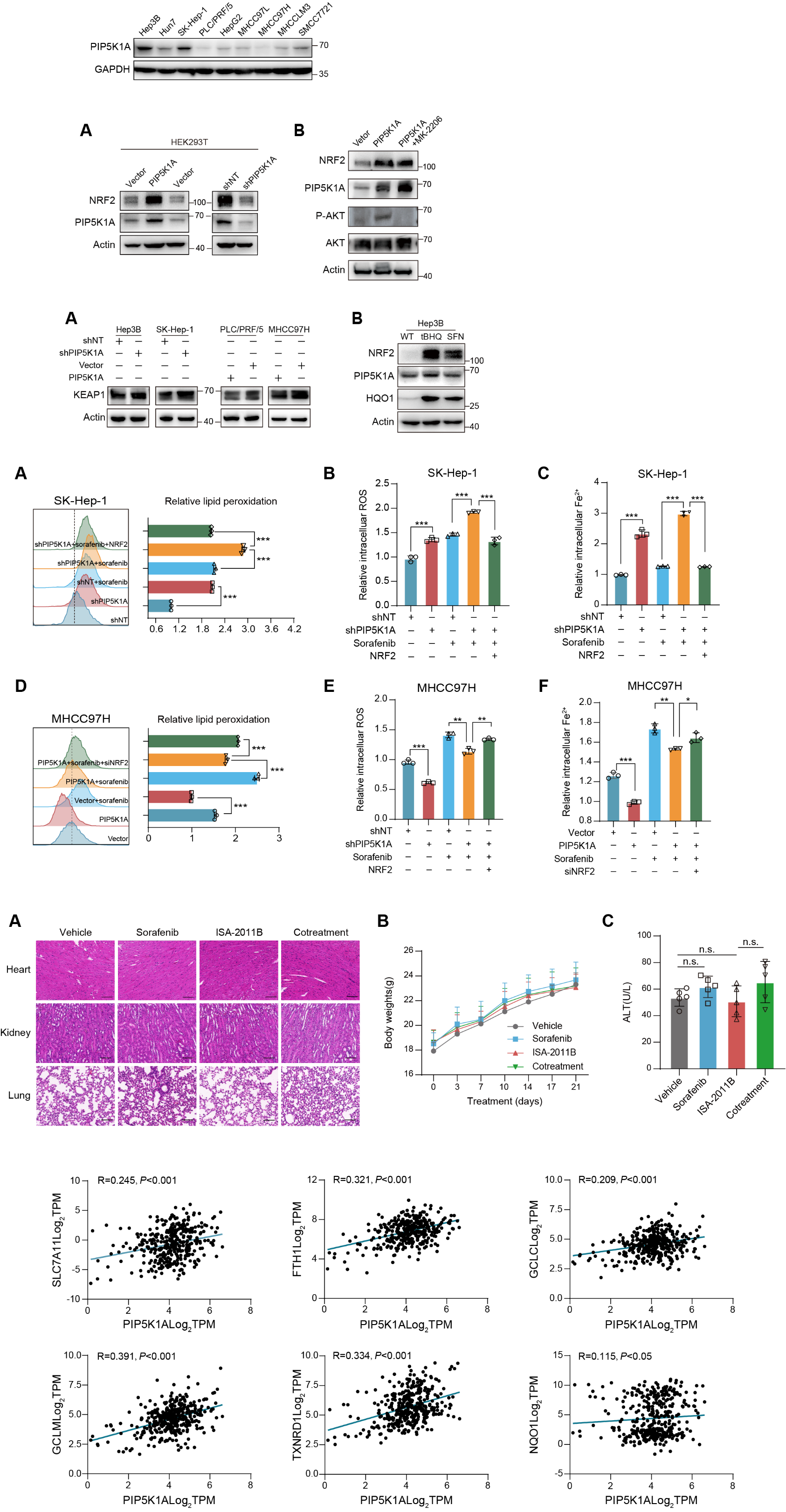
**

**Figure S4.** PIP5K1A regulates NRF2 expression in a PI3K/AKT independent way. A) NRF2 protein expression in HEK293T cells following transfection with either PIP5K1A overexpression plasmids, PIP5K1A knockdown constructs, or their respective control vectors. B) Western blotting of NRF2, PIP5K1A, P-AKT and total AKT in MHCC97H-Vector and MHCC97H-PIP5K1A cells treated with or without MK-2206 (10 μm) for 24 h.

**
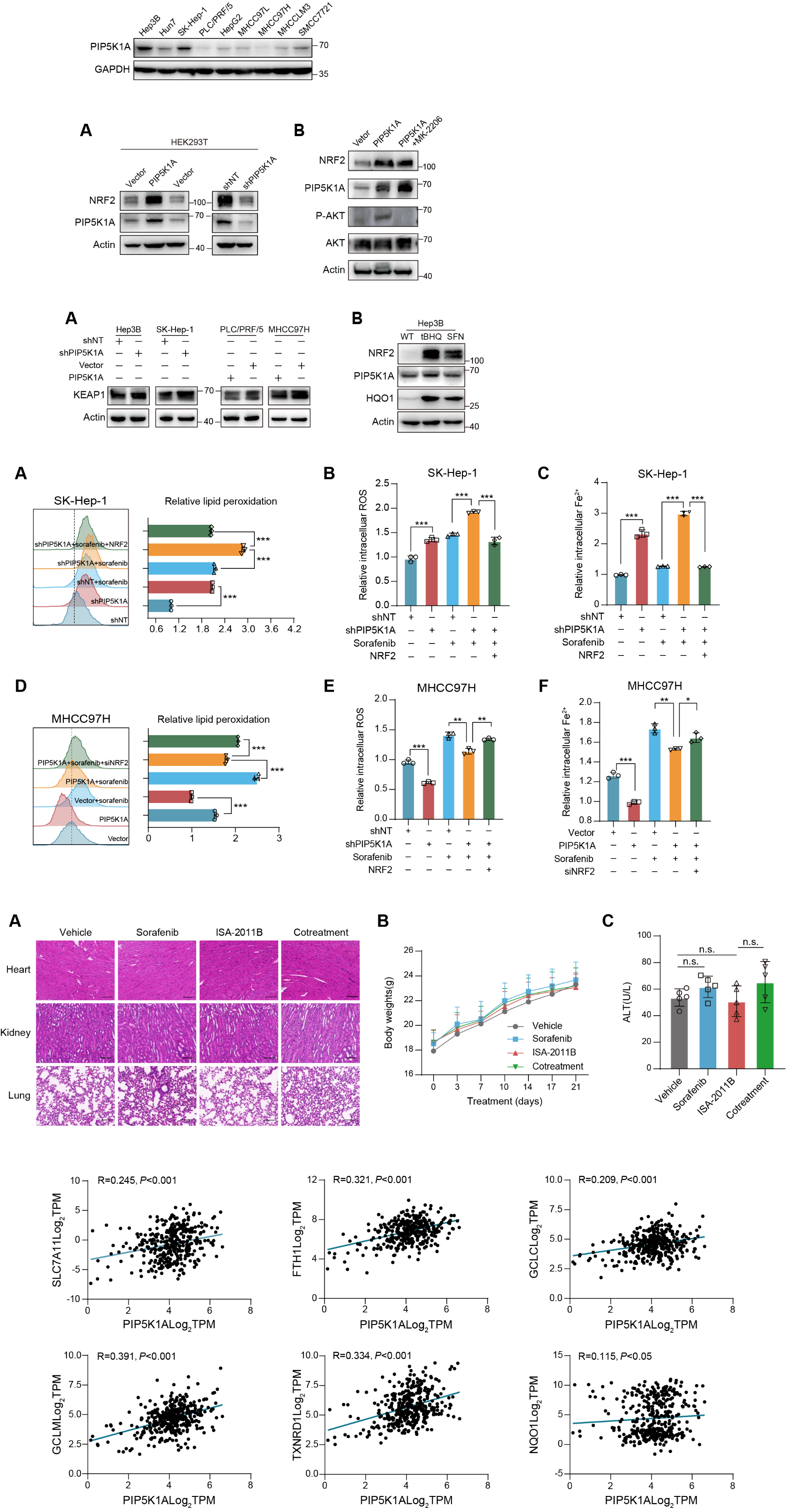
**

**Figure S5.** PIP5K1A does not affect KEAP1 protein levels and is not regulated by NRF2. A) Western blotting of KEAP1 expression following PIP5K1A knockdown or overexpression. B) Protein levels of NRF, PIP5K1A and NQO1 in Hep3B cells treated with tBHQ (50 μm) or SFN (5 μm) for 24h. Abbreviations: tBHQ, tert-butylhydroquinone; SFN, sulforaphane.


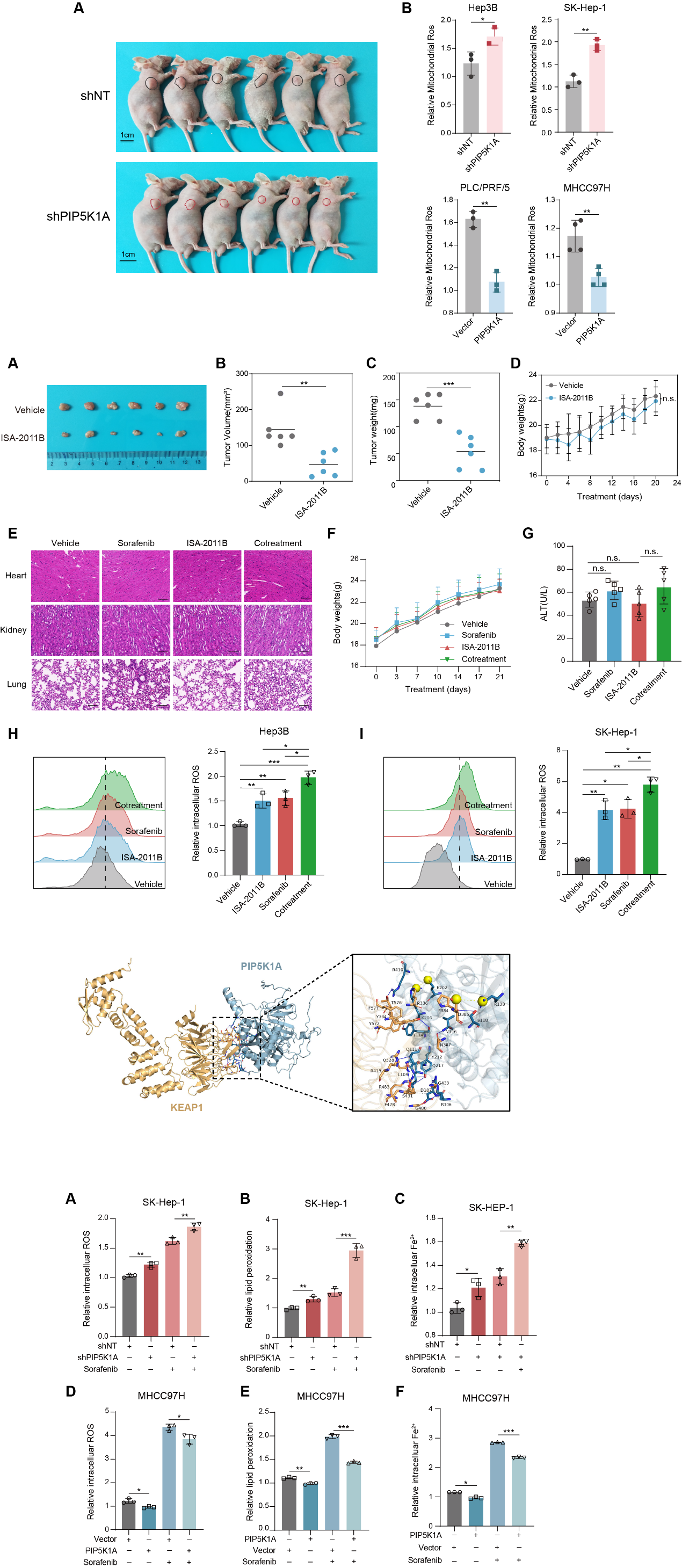


**Figure S6.** The most optimal predicted binding model of PIP5K1A to KEAP, and

the specific interacting residues involved in the interaction of PII5K1A and KEAP1 were shown.

**
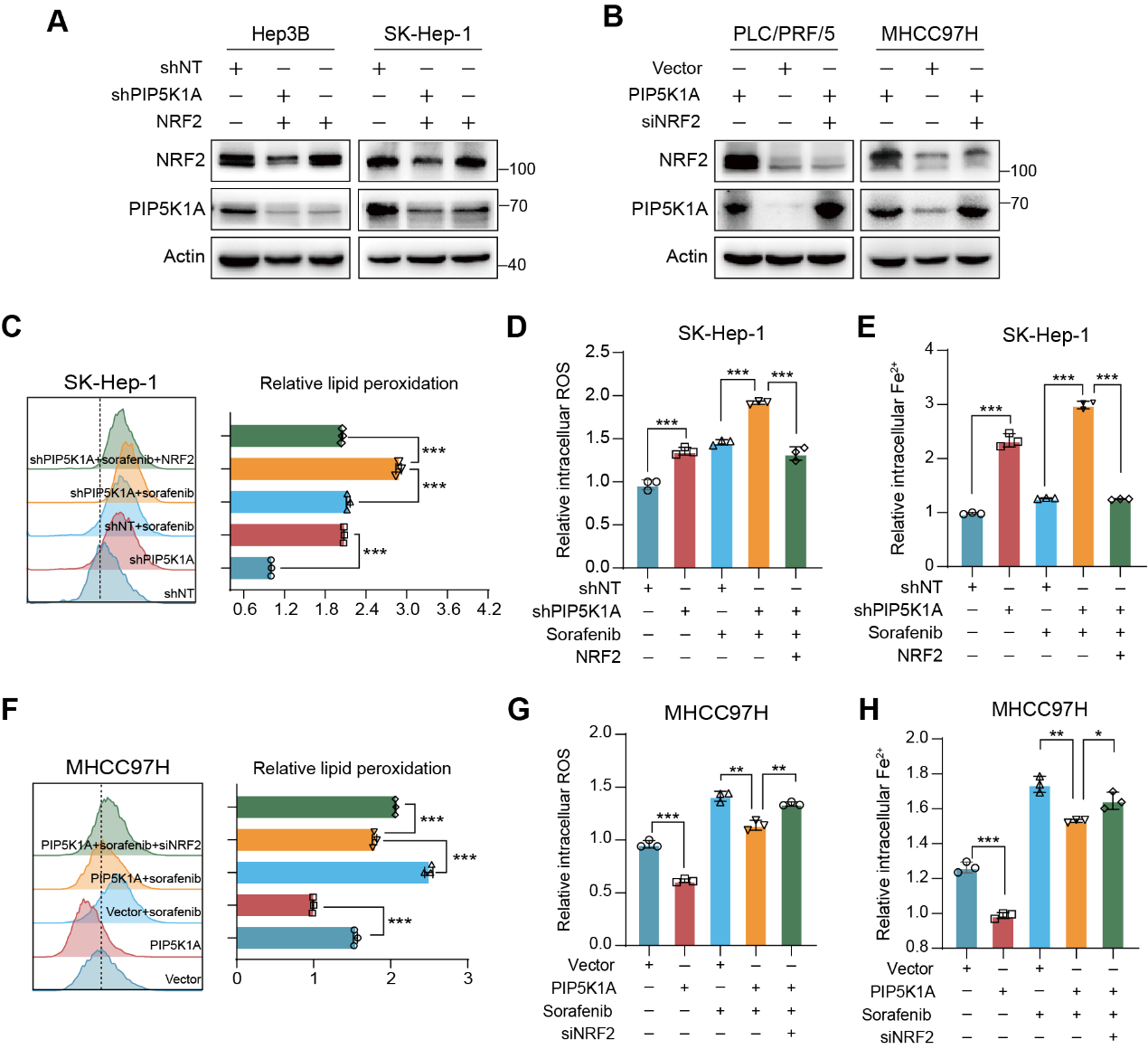
**

**Figure S7.** PIP5K1A inhibits sorafenib-induced ferroptosis by activating NRF2 in HCC cells. A) Western blot analysis of NRF2 and PIP5K1A expression in control and PIP5K1A-knockdown cells transfected with or without NRF2 plasmids for 24h. B) Western blot analysis of NRF2 and PIP5K1A expression in vector and PIP5K1A-overpressing cells transfected with or without NRF2 siRNA for 24h. C-E) Control and PIP5K1A-knockdown HCC cells were transfected with or without NRF2 plasmids, and treated with either DMSO or sorafenib (5 µm) for 24 h. Lipid ROS, total ROS and Fe^2+^ levels were then detected by flow cytometry. F-H) Vector and PIP5K1A-overpressing cells were transfected with or without NRF2 siRNA and treated with either DMSO or sorafenib (5 µm) for 24 h. Lipid ROS, total ROS and Fe^2+^ levels were then detected by flow cytometry. All data are presented as the means ± SD (n=3). **P* < 0.05, ***P* < 0.01, ****P* < 0.001. Abbreviations: ROS: reactive oxygen species.

**
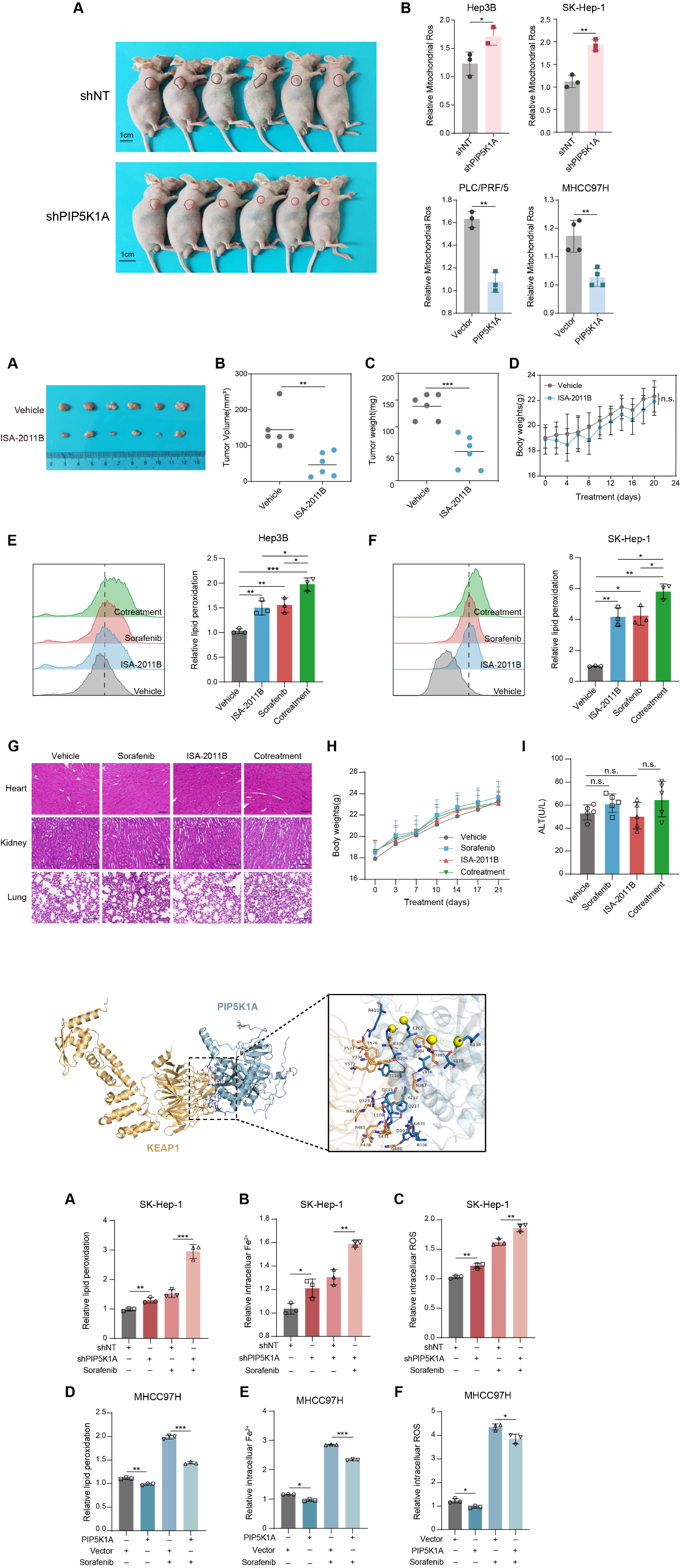
**

**Figure S8.** ISA-2011B inhibits HCC growth without causing additional toxicity when combined with sorafenib in *vivo*. A) Representative image of subcutaneous xenografts models inoculated with SK-Hep-1 cells. Seven days post-inoculation, mice received either control or ISA-2011B (40 mg/kg) through injection intraperitoneally once every other day for 14 days (n=6). B-C) Quantification of tumor volume and weight at the end of experiments. D) Body weights of the tumor-bearing mice measured every four days. E-F) lipid ROS levels in Hep3B and SK-Hep-1 cells treated with control, ISA-2011B (50 µM), Sorafenib (8 µM), or ISA-2011B (50 µM) + Sorafenib (8 µM) for 24 hours. G) Representative H&E staining of heart, kidney and lung tissues form all treatment groups. Scale bar: 200 μm. H) Body weight measurements of tumor-bearing mice performed twice weekly across treatment groups. I) Serum levels of ALT were detected in all treatment groups. Data are presented as the means ± SD. *, *P* < 0.05; **, *P* < 0.01; ***, *P* < 0.001; n.s., not significant. Abbreviations: ALT, Alanine aminotransferase.

**
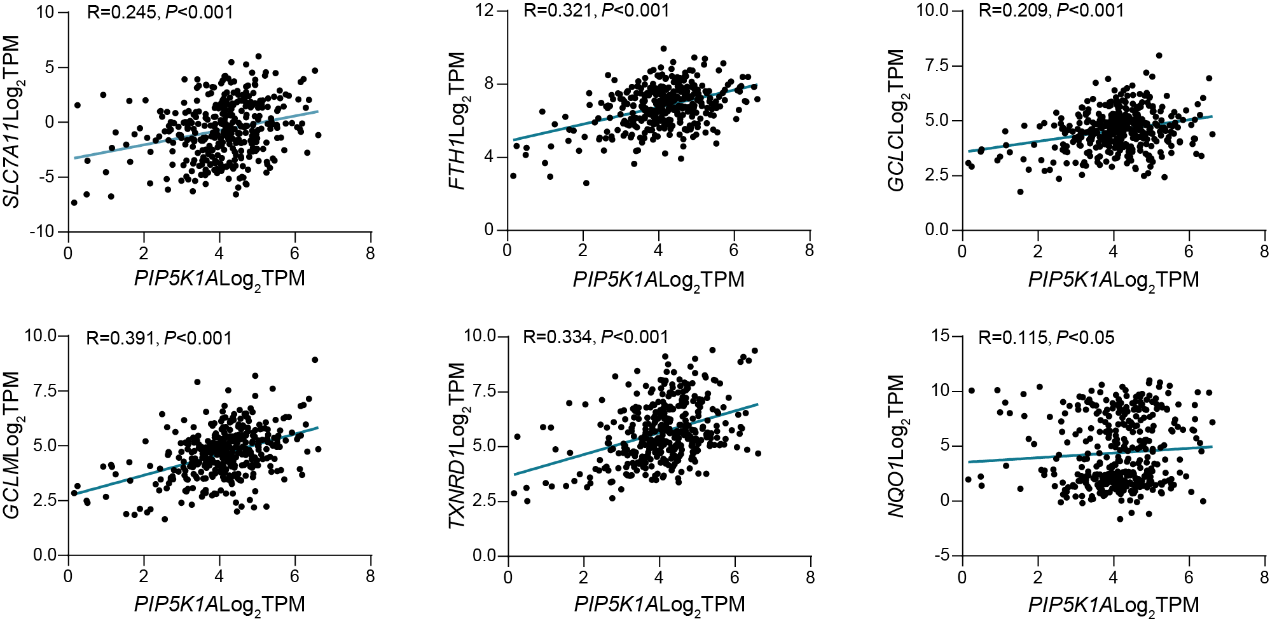
**

**Figure S9.** PIP5K1A expression positively correlates with expression of NRF2 target genes. In the TCGA HCC cohort, the transcriptional level of *PIP5K1A* was positively correlated with mRNA expression of NRF2 target genes (*SLC7A11, FTH1, GCLC, GCLM, TXNRD1 and NQO1*) involved in the ferroptosis signaling pathway.

**Table S1. Clinicopathologic characteristics of 354 HCC patients.**

| Characteristics |  | Patients |
| --- | --- | --- |
|  |  | Number (%) |
| Age, years | ≤50 | 141(39.8) |
|  | >50 | 213(60.2) |
| Gender | Female | 60(16.9) |
|  | Male | 294(83.1) |
| HbsAg | Negative | 57(16.1) |
|  | Positive | 297(83.9) |
| AFP, ng/ml | ≤20 | 130(36.7) |
|  | >20 | 224(63.3) |
| ALT, U/L | ≤40 | 167(47.2) |
|  | >40 | 187(52.8) |
| γ-GT, U/L | ≤54 | 183(51.7) |
|  | >54 | 171(48.3) |
| Liver cirrhosis | No | 68(19.2) |
|  | Yes | 286(80.8) |
| Tumor number | Single | 295(83.3) |
|  | Multiple | 59(16.7) |
| Tumor size, cm | ≤5 | 214(60.5) |
|  | >5 | 140(39.5) |
| Tumor encapsulation | Complete | 195(55.1) |
|  | None | 159(44.9) |
| Tumor differentiation | I-II | 266(75.1) |
|  | III-IV | 88(24.9) |
| Microvascular invasion | Absent | 241(68.1) |
|  | Present | 113(31.9) |
| BCLC stage | 0 + A | 195(55.1) |
|  | B + C | 159(44.9) |

**Abbreviations:** HCC, hepatocellular carcinoma; HBsAg, hepatitis B surface antigen; AFP, α-fetoprotein; ALT: alanine aminotransferase; γ-GT, γ-glutamyl transferase; BCLC, Barcelona Clinic Liver Cancer.

**Table S2. Primary antibodies used in this study**

| Antibody | Dilutions for WB | Dilutions for IHC | Dilutions for IF | Dilutions for IP | Company |
| --- | --- | --- | --- | --- | --- |
| PIP5K1A | 1:1000 |  |  |  | CST (#9693) |
| PIP5K1A |  | 1:200 | 1:100 | 1:200 | Proteintech (15713-1-AP) |
| NRF2 | 1:1000 |  | 1:200 |  | CST (#12721) |
| NRF2 |  | 1:200 |  |  | Proteintech (16396-1-AP) |
| KEAP1 | 1:1000 |  | 1:100 | 1:200 | CST (#8047) |
| Flag | 1:1000 |  |  | 1:200 | Abclonal (AE005) |
| His | 1:1000 |  |  | 1:200 | Abclonal (AE003) |
| Myc | 1:1000 |  |  | 1:200 | Abclonal (AE010) |
| p-AKT | 1:1000 |  |  |  | Abclonal (AP0637) |
| AKT | 1:1000 |  |  |  | Abclonal (A18675) |
| Ubiquitin | 1:1000 |  |  |  | CST (#3936) |
| NQO1 | 1:1000 |  |  |  | CST (#62262) |
| Lamin B1 | 1:1000 |  |  |  | Proteintech (12987-1-AP) |
| 4-HNE |  | 1:200 |  |  | Abcam (AB205718) |
| SLC7A11 |  | 1:200 |  |  | Abcam (AB205721) |
| GPX4 |  | 1:400 |  |  | Abcam (AB205722) |
| Actin | 1:1000 |  |  |  | Beyotime (AF0003) |
| GAPDH | 1:1000 |  |  |  | Beyotime (AF0006) |

**Table S3. Sequences of primers used for qRT-PCR**

| Primer | Sequence（5' to 3'） | NCBI Reference Sequence |
| --- | --- | --- |
| *PIP5K1A*-hum-F | AGATTCCCTGCGTTCACCTT | NM_001135637.2 |
| *PIP5K1A*-hum-R | TGAGGCTTTGCGCTTAATGG |  |
| *NFE2L2*-hum-F | CAGTCAGCGACGGAAAGAGTA | [NM_006164.5](http://www.ncbi.nlm.nih.gov/nuccore/NM_006164.5) |
| *NFE2L2*-hum-R | TGTGGGCAACCTGGGAGTAG |  |
| *NQO1*-hum-F | GCTGGTTTGAGCGAGTGTTC | NM_000903.3 |
| *NQO1*-hum-R | GCCTTCTTACTCCGGAAGGG |  |
| *FTH1*-hum-F | CCCCCATTTGTGTGACTTCAT | NM_002032.3 |
| *FTH1*-hum-R | GCCCGAGGCTTAGCTTTCATT |  |
| *SLC7A11*-hum-F | TGCTGGGCTGATTTATCTTCG | [NM_014331.4](http://www.ncbi.nlm.nih.gov/nuccore/NM_014331.4) |
| *SLC7A11*-hum-R | GAAAGGGCAACCATGAAGAGG |  |
| *GCLC*-hum-F | TTAGGCTGTCCTGGGTTCAC | [NM_001498.4](http://www.ncbi.nlm.nih.gov/nuccore/NM_001498.4) |
| *GCLC*-hum-R | TCGCTCCTCCCGAGTTCTAT |  |
| *GPX4*-hum-F | GAGGCAAGACCGAAGTAAACTAC | NM_002085.5 |
| *GPX4*-hum-R | CCGAACTGGTTACACGGGAA |  |
| *GCLM*-hum-F | TGTCTTGGAATGCACTGTATCTC | [NM_002061.4](http://www.ncbi.nlm.nih.gov/nuccore/NM_002061.4) |
| *GCLM*-hum-R | CCCAGTAAGGCTGTAAATGCTC |  |
| *CAT*-hum-F | GTGAACTGTCCCTACCGTGC | NM_001752.4 |
| *CAT*-hum-R | AGGGCAGAAGGCTGTTGTTC |  |
| *TXNRD1*-hum-F | TAGGACAAGCCCTGCAAGACT | [NM_001093771.3](http://www.ncbi.nlm.nih.gov/nuccore/NM_001093771.3) |
| *TXNRD1*-hum-R | CCCCAATTCAAAGAGCCAATGT |  |
| *ACTB*-hum-F | CATGTACGTTGCTATCCAGGC | NM_001101.5 |
| *ACTB*-hum-R | CTCCTTAATGTCACGCACGAT |  |
